# YY1-induced upregulation of LncRNA-ARAP1-AS2 and ARAP1 promotes diabetic kidney fibrosis *via* aberrant glycolysis associated with EGFR/PKM2/HIF-1α pathway

**DOI:** 10.3389/fphar.2023.1069348

**Published:** 2023-02-15

**Authors:** Xin Li, Tian-Kui Ma, Min Wang, Xiao-Dan Zhang, Tian-Yan Liu, Yue Liu, Zhao-Hui Huang, Yong-Hong Zhu, Shuang Zhang, Li Yin, Yan-Yan Xu, Hong Ding, Cong Liu, Hang Shi, Qiu-Ling Fan

**Affiliations:** ^1^ Department of Nephrology, First Hospital of China Medical University, Shenyang, China; ^2^ Department of Nephrology, Fourth Hospital of China Medical University, Shenyang, China; ^3^ Department of General Surgery, First Hospital of Harbin Medical University, Harbin, China; ^4^ Department of Intensive Care Unit, Sun Yat-sen Memorial Hospital of Sun Yat-sen University, Guangzhou, China; ^5^ Department of Nephrology, Shanghai General Hospital, Shanghai Jiao Tong University School of Medicine, Shanghai, China

**Keywords:** LncRNA-ARAP1-AS2, ARAP1, diabetic kidney disease, HIF-1α, glycolysis, fibrosis, PKM2

## Abstract

**Objectives:** Dimeric pyruvate kinase (PK) M2 (PKM2) plays an important role in promoting the accumulation of hypoxia-inducible factor (HIF)-1α, mediating aberrant glycolysis and inducing fibrosis in diabetic kidney disease (DKD). The aim of this work was to dissect a novel regulatory mechanism of Yin and Yang 1 (YY1) on lncRNA-ARAP1-AS2/ARAP1 to regulate EGFR/PKM2/HIF-1α pathway and glycolysis in DKD.

**Materials and methods:** We used adeno-associated virus (AAV)-ARAP1 shRNA to knocked down ARAP1 in diabetic mice and overexpressed or knocked down YY1, ARAP1-AS2 and ARAP1 expression in human glomerular mesangial cells. Gene levels were assessed by Western blotting, RT-qPCR, immunofluorescence staining and immunohistochemistry. Molecular interactions were determined by RNA pull-down, co-immunoprecipitation, ubiquitination assay and dual-luciferase reporter analysis.

**Results:** YY1, ARAP1-AS2, ARAP1, HIF-1α, glycolysis and fibrosis genes expressions were upregulated and ARAP1 knockdown could inhibit dimeric PKM2 expression and partly restore tetrameric PKM2 formation, while downregulate HIF-1α accumulation and aberrant glycolysis and fibrosis in *in-vivo* and *in-vitro* DKD models. ARAP1 knockdown attenuates renal injury and renal dysfunction in diabetic mice. ARAP1 maintains EGFR overactivation *in-vivo* and *in-vitro* DKD models. Mechanistically, YY1 transcriptionally upregulates ARAP1-AS2 and indirectly regulates ARAP1 and subsequently promotes EGFR activation, HIF-1α accumulation and aberrant glycolysis and fibrosis.

**Conclusion:** Our results first highlight the role of the novel regulatory mechanism of YY1 on ARAP1-AS2 and ARAP1 in promoting aberrant glycolysis and fibrosis by EGFR/PKM2/HIF-1α pathway in DKD and provide potential therapeutic strategies for DKD treatments.

## 1 Introduction

Diabetic kidney disease (DKD) is an important cause of end-stage renal disease. Current treatment strategies include 1) blood glucose control such as DPP-4 inhibitor linagliptin and SGLT-2 inhibitor empagliflozin (Srivastava et al., 2020a; Pugliese et al., 2020; Herrington et al., 2023), 2) blood pressure control such as Ang-II receptor blockers (ARBs) or angiotensin converting enzyme (ACE) inhibitors (Roett et al., 2012) and 3) blood lipid control (Srivastava et al., 2014), as well as new potential therapeutic strategies as 1) SIRT3 activation, 2) glycolysis inhibitors, 3) Rho kinase (ROCK) inhibitor Fasudil, 4) endogenous antifibrotic peptide N-acetyl-seryl-aspartyl-lysyl-proline (AcSDKP), which is associated with fibroblast growth factor receptor 1 (FGFR1) phosphorylation, 5) endothelial and podocyte glucocorticoid receptor (GR) delivery and so on (Kitada et al., 2013; Srivastava et al., 2016; Srivastava et al., 2018; Morita and Kanasaki, 2020; Srivastava et al., 2021a; Srivastava et al., 2021b; Wang et al., 2021). However, current treatments cannot completely stop or delay disease progression. Therefore, in-depth study of the pathogenesis of DKD and search for targets and biomarkers of accurate diagnosis and early individualized treatment have become urgent public health issues to be solved.

Glomerular mesangial cells (GMCs) are the main cell types responsible for the generation of extracellular matrix (ECM) (Qiao et al., 2019). The massive deposition of ECM is a critical indicator of diabetic renal fibrosis, closely associated with activated fibroblasts, which can be produced by epithelial cells *via* epithelial-to-mesenchymal transition (EMT), by bone marrow-derived M2 phenotype macrophages *via* macrophage-to-mesenchymal transition (MMT), by endothelial cells *via* endothelial-to-mesenchymal transition (EndMT). And TGF-β, BMP, Wnt and Sonic Hedgehog signaling play a crucial role in the activation of mesenchymal transition processes (Srivastava et al., 2020b). The fibrosis in kidney has been shown to be associated with aberrant glycolysis, known as the Warburg effect (Liu et al., 2021). Such aberrant glycolysis, which is characterized by the accumulation of hypoxia-inducible factor-1α (HIF-1α), promotes the expression of fibrotic genes and the expansion of mesangial matrix (Jia et al., 2022). However, in DKD, the molecular mechanism of aberrant glycolysis in GMCs has not been fully elucidated.

The epidermal growth factor receptor (EGFR) enhances the production of the key ECM components collagen I (COL I), collagen IV (COL IV), and fibronectin (FN) in GMCs, aggravating renal fibrosis and glomerulosclerosis in DKD (Uchiyama-Tanaka et al., 2002; Wu et al., 2009a; Wu et al., 2009b; Taniguchi et al., 2013). EGFR activation mediates HIF-1α activation and subsequent the induction of aberrant glycolysis, which finally promoting renal fibrosis in DKD (Yu et al., 2012; Li et al., 2015). Pyruvate kinase (PK) M2 (PKM2), the critical enzyme in glycolysis, can be aggregated into tetramer and dimer forms, and PKM2 in the dimer state can enter the nucleus to regulate gene expression (Azoitei et al., 2016; Zhang et al., 2019a). The transformation between PKM2 dimer and tetramer plays an important role in many diseases, such as DKD and breast cancer (Liu et al., 2021; Wu et al., 2022). EGFR activation can increase dimer PKM2 expression and induce translocation of dimer PKM2 into the nucleus, where PKM2 acts both as a protein kinase and a transcriptional coactivator for HIF-1α in tumor tissues (He et al., 2014; Azoitei et al., 2016). In the fibrotic kidney of diabetic mice, PKM2 tetramer formation is suppressed, while the PKM2 dimer expression is induced and enter the nucleus and directly interacts with HIF-1α, promoting HIF-1α transactivation as well as the expression of its downstream glycolytic genes. Activation of tetrameric PKM2 formation can reduce the entry of PKM2 dimer form into the nuclear and activation of HIF-1α, alleviate abnormal glycolysis and renal fibrosis, and play a role in renal protection (Luo et al., 2011; Qi et al., 2017; Sizemore et al., 2018; Liu et al., 2021). We have reported that natural antisense lncRNA-ARAP1-AS2 can interact with its sense target gene ARAP1, a type 2 diabetes susceptibility gene product, which finally contributing to the fibrosis process through maintaining the EGFR persistent transactivation in human proximal tubular cells (HK-2) exposed to the high glucose condition (Yang et al., 2019a; Li et al., 2020a; Li et al., 2020b). Nevertheless, the relationship between ARAP1-AS2/ARAP1, EGFR activation, PKM2 and HIF-1α activation as well as aberrant glycolysis in DKD is currently unknown.

Non-coding RNAs (ncRNAs) are a kind of functional RNA molecule, including microRNA (miRNA), circular RNA (circRNA) and long non-coding RNA (lncRNA), playing a pivotal in regulating MMT, EMT and EndMT progress in DKD (Srivastava et al., 2013; Srivastava et al., 2020b; Peng et al., 2021; Zhang et al., 2021). miRNA have been intensely investigated and miRNA crosstalk in the kidney is found to regulate various disease progresses, such as MMT, EMT and EndMT (Srivastava et al., 2013). However, the functional role of lncRNAs remains poorly understood. Evidence have demonstrated that lncRNAs are involved in the pathophysiology, such as pathologic processes in mesangial cells, podocytes, oxidative stress, EMT, EndMT and actions on miRNAs in DKD (Srivastava et al., 2021c). Yin and Yang 1 (YY1), a novel therapeutic target for early DKD-associated tubulointerstitial fibrosis (Yang et al., 2022), mainly distributed in the nucleus and directly or indirectly binds to gene promoters to activate or repress expression *via* chromatin remodeling or histone modification in DKD (Yang et al., 2019b). YY1 is also known to interact with lncRNA (An and Ding, 2021). To date, the regulatory mechanism of YY1 on ARAP1-AS2 is unclear in DKD.

Currently, the involvement of ARAP1-AS2/ARAP1 during the pathogenesis of DKD in diabetic mice and renal cell populations other than human proximal tubular cells remains unclear. In this study, we investigated the hypothesis that ARAP1 can aggravate ECM accumulation and renal dysfunction and glomerulosclerosis in diabetic db/db mice as well as in GMCs exposed to high glucose through aberrant glycolysis, associated with HIF-1α accumulation led by increased PKM dimer formation as well as decreased PKM tetramer formation through maintaining the EGFR persistent activation. We also explored the prediction that YY1 targets the promoter of ARAP1-AS2 and positively regulates ARAP1-AS2 transcriptional activity in DKD. We provide the first evidence for YY1 regulate EGFR persistent activation, transformation between PKM2 dimer and tetramer, HIF-1α accumulation and aberrant glycolysis and fibrosis in DKD through the ARAP1-AS2/ARAP1 axis. These results further highlight the possibility of pursuing the YY1/ARAP1-AS2/ARAP1 axis as a promising therapeutic target for DKD treatment.

## 2 Materials and methods

### 2.1 Cell culture

Human renal mesangial cells (HRMCs) were purchased from ScienCell Research (San Diego, United States), and the mouse mesangial cell (MMC) line SV40 MES-13 was purchased from the American Type Culture Collection (ATCC). All cells were cultured in DMEM (Gibco, United States) containing 10% fetal bovine serum (Gibco, Australia), streptomycin (100 μg/mL) and penicillin (100 U/mL) at 37°C and 5% CO_2_. Standard medium containing 5 mM D-glucose was used for the normal glucose (NG) condition. We added 20 mM glucose (yielding a final concentration of 25 mM) or mannitol for the high glucose (HG) condition or osmotic control (MA) and stimulated the cells for 48 h. The concentration was chosen based on our previous studies (Lu et al., 2015; Wang et al., 2018a; Ma et al., 2019).

### 2.2 Plasmids, siRNAs, the EGFR tyrosine kinase inhibitor AG1478 and transfection

We used the same methods as in our previous reports for synthesizing shRNA targeting human ARAP1 and siRNA targeting human ARAP1-AS2 (Li et al., 2020a; Li et al., 2020b). The siRNAs targeting mouse ARAP1, overexpression plasmids and siRNAs targeting human YY1 and overexpression plasmids targeting human ARAP1were purchased from Sangon Biotechnology (Shanghai, China). The specific EGFR tyrosine kinase inhibitor AG1478 (MedChemExpress LLC) was used to suppress the phosphorylation of EGFR in HRMCs. Powdered AG1478 was dissolved in DMSO, and the final concentration of AG1478 administered to HRMCs was 10 μmol/L. Cells were exposed to AG1478 for 48 h. Transfection methods, cell lines created by transfection and their names are described in the Supplementary Material and Methods.

### 2.3 RNA pulldown sequencing (pulldown-seq)

RNA pulldown-seq was performed as our group reported previously (Li et al., 2020b) and the details are provided in the Supplementary Material and Methods.

### 2.4 RNA pulldown assay

Biotin-labelled sense or antisense oligos of ARAP1-AS2 were incubated with HRMCs’ lysates for 2 h. The complex was pulled down by streptavidin-coated magnetic beads (M-280 Dynabeads; Invitrogen). The amount of YY1 mRNA was measured by qRT-PCR.

### 2.5 Functional enrichment analysis

Genes enriched in the RNA pulldown-seq assay were evaluated by functional and enrichment analysis using GO (Gene Ontology 2004) (http://geneontology.org/) and KEGG (http://www.genome.jp/kegg/) databases (*p* < 0.05, fold enrichment>1.5). For GO analyses, enriched biological processes (BPs), molecular functions (MFs), and cellular components (CCs) were assessed.

### 2.6 Dual-luciferase reporter analysis

For gene promoter luciferase analysis, HRMCs were plated into 24-well plates. The possible YY1 binding sites in the ARAP1-AS2 promoter sequence were predicted by the JASPAR tool. ARAP1-AS2 WT/Mut plasmids were constructed by inserting ARAP1-AS2 promoter fragments containing wild-type (Wt) or mutant (Mut) YY1 binding sites into the pGL4.10 reporter vector (SyngenTech, Beijing, China, Supplementary Figure S1), and the plasmids were co-transfected into cells with siYY1 or siNC. A reporter gene assay was conducted after 48 h using a Dual Luciferase Assay System (Promega).

### 2.7 Animals

All animal procedures were approved by the Institutional Animal Care and Use Committee of China Medical University, and ethical approval was obtained from the Ethics Committee of China Medical University (number: CMU2019222). Ten-week-old diabetic male db/db mice (C57BLKS/J-leprdb/leprdb, *n* = 30) and normal control male db/m mice (C57BLKS/J-leprdb/+, *n* = 10) were purchased from the Model Animal Research Center of Nanjing University (Nanjing, China). Mice were housed at 22°C ± 2.0°C under a 12-h:12-h light/dark cycle under 50% ± 20% humidity at the Laboratory Animal Center of China Medical University and fed food and water *ad libitum*. We collected tail vein blood when the mice were 12 weeks of age and measured fasting blood glucose levels to confirm spontaneous hyperglycemia. Diabetes was defined as a blood glucose level greater than 16.7 mM.

### 2.8 Knockdown of ARAP1 in diabetic mice

Adeno-associated virus (AAV) 2/9-shARAP1 and AAV2/9-NC were purchased from Sangon Biotechnology (Shanghai, China). The viral vector construction framework for AAV2/9-shARAP1 is described in Supplementary Table S1, and the AAV vector backbone are provided in Supplementary Figure S2. Experimental groups in animal studies and their designations are described in the Supplementary Material and Methods. The mice were fasted for more than 8 h before measuring blood glucose and urine. Body weight (BW) and fasting blood glucose levels were measured once every 2 weeks for 8 successive weeks. Individual metabolic cages were used to collect mouse urine samples every 2–3 weeks. All mice were euthanized at 20 weeks, and blood and kidney tissue samples were collected for subsequent analyses.

### 2.9 Serum and urine biochemistry

A VITROS 950 automatic biochemical analyzer (Johnson & Johnson, NJ) was used to measure serum creatinine and blood urea nitrogen (BUN). The ELISA for mouse urinary albumin and neutrophil gelatinase-associated lipocalin (NGAL) was performed as described previously by our team (Zhang et al., 2019b). Urinary creatinine concentrations were measured with a creatinine assay kit (NJJCBIO, C011-2, China). The UACR was calculated as the ratio of urinary albumin concentration and creatinine concentration (mg/g). Urine samples were collected when the mice were 12 weeks of age and were considered to have DKD when the UACR was greater than 3 mg/mmol.

### 2.10 Kidney pathology

Mouse kidney tissues were fixed in 4% paraformaldehyde and embedded in paraffin. Sections (3 μm) were obtained and stained with hematoxylin-eosin (H-E), Masson’s trichrome, and periodic acid-Schiff (PAS) according to the manufacturer’s instructions. The slides were examined under a Leica microscope for subsequent analysis. The glomerulosclerosis index (GSI) was calculated as described previously by our group, and the details are provided in the Supplementary Material and Methods (Ma et al., 2022). To obtain GSI, 3 experienced renal pathologists independently scored 30–50 glomeruli, and the average value was used as the final data (Ma et al., 2001).

### 2.11 Isolation of mouse glomeruli

Mouse glomeruli were isolated as previously described, and the details are provided in the Supplementary Material and Methods (Zhao et al., 2018).

### 2.12 Immunohistochemical (IHC) analysis

Detailed description of methods for IHC is provided in the Supplementary Material and Methods. The primary antibodies used in this study and the dilution ratio are provided in Supplementary Table S2.

### 2.13 Immunofluorescence staining

#### 2.13.1 For HRMC

The process was provided in Supplementary Material and Methods. The primary antibodies used were anti-HIF-1α (1:200, NB100-123, Novus, United States).

#### 2.13.2 For tissue sections

The process was provided in Supplementary Material and Methods. The primary antibodies used were anti-ARAP1 (1:50, sc-393138, Santa Cruz Biotechnology) and anti-HIF-1α (1:200, NB100-123, Novus, United States).

### 2.14 qRT-PCR

Detailed description of methods is provided in the Supplementary Material and Methods. Primer sequences are provided in Supplementary Table S3.

### 2.15 Western blot analysis

Detailed description of methods is provided in the Supplementary Material and Methods. The antibody dilution ratios are provided in Supplementary Table S4.

### 2.16 Cell counting Kit-8 (CCK-8) assay

Twenty-five hundred (2.5 × 10^3^) cells per well were seeded in a 96-well plate. Ten microliters of CCK-8 reagent (Dojindo) were added to the medium in each well on Days 0, 1, 2 and 3, and the 96-well plate was maintained at 37°C for 2 h. Then, the absorbance was measured using a microplate reader (BioTek Instruments).

### 2.17 Coimmunoprecipitation (Co-IP)

#### 2.17.1 For HRMC

Co-IP experiments for cell protein lysates were performed as previously described (Li et al., 2020b). The primary antibodies used were as follows: anti-ARAP1 (sc-393138, Santa Cruz Biotechnology), anti-CIN85 (#12304, Cell Signaling Technology) and corresponding control mouse IgG or rabbit IgG (B900620, B900610, Proteintech).

#### 2.17.2 For mouse glomeruli

We performed Co-IP experiments using a Dynabeads Protein G Immunoprecipitation kit (Invitrogen, United States) according to the manufacturer’s instructions, and detailed description of the methods is provided in the Supplementary Material and Methods.

### 2.18 Ubiquitination assay

#### 2.18.1 For HRMC

Ubiquitination experiments for cell protein lysates were performed as previously described (Li et al., 2020b). The primary antibodies used for Western blotting were as follows: anti-EGFR antibody (ab52894, Abcam), anti-ubiquitin (#3933, Cell Signaling Technology) and corresponding control rabbit IgG (B900610, Proteintech).

#### 2.18.2 For mouse glomeruli

For EGFR ubiquitination, detailed description of the methods is provided in the Supplementary Material and Methods.

### 2.19 Cross-linking assay

Mouse glomeruli or HRMCs were lysed with sodium phosphate buffer (pH 7.3) containing 0.5% Triton X-100 and protease inhibitor for 30 min at 4°C. The lysates were centrifuged at 20,000 rpm for 30 min at 4°C, and the supernatants were collected. The supernatants were then treated with 0.01% glutaraldehyde for 5 min at 37°C and were terminated by using 50 mM Tris-Cl (pH 8.0). These samples were separated by 10% SDS-PAGE and analyzed by Western blot with anti-PKM2 antibody (15822-1-AP, Proteintech).

### 2.20 Statistical analyses

Data are presented as the mean ± SD. Student’s t-test and ANOVA with *post hoc* tests were used to analyze the significance of differences between groups. *p* < 0.05 was considered statistically significant. Statistical analysis of the results was performed with SPSS 26.0 software (IBM). Each experiment was done at least for repeated three times independently.

## 3 Results

### 3.1 Knockdown of ARAP1 reduces renal PKM2 dimer expression, HIF-1α accumulation, fibrogenesis and aberrant glycolysis accompanied by restoring tetrameric PKM2 formation in diabetic mice

**FIGURE 1:**
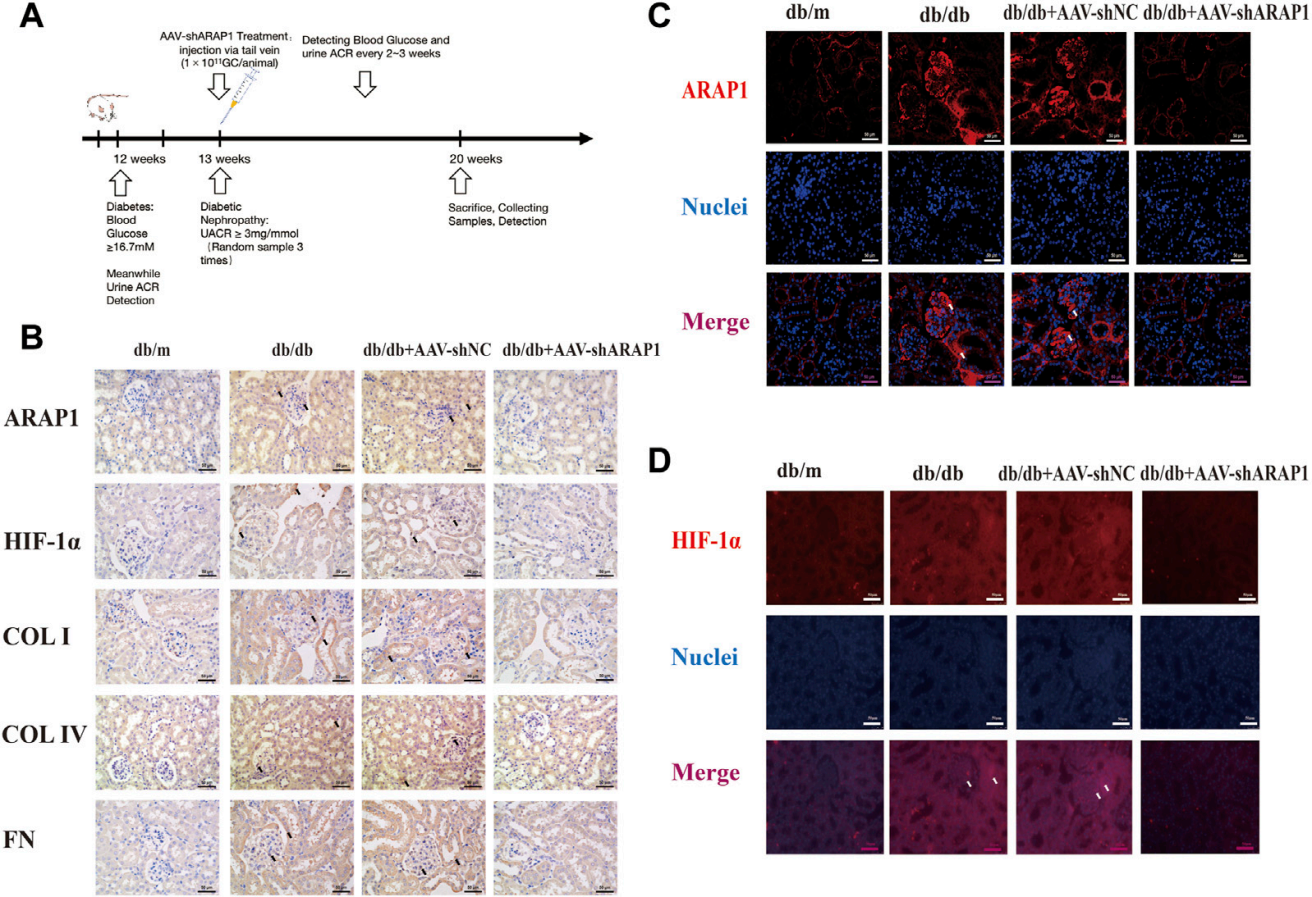
ARAP1 knockdown reduces renal HIF-1α accumulation, fibrogenesis and aberrant glycolysis in diabetic mouse **(A)** Experimental flow chart. **(B)** IHC detection of ARAP1, HIF-1α, COL I, COL IV and FN in kidney tissues from db/m, db/db, db/db + AAV-shNC and db/db + AAV-shARAP1 mice (×400). Bar = 50 μm. **(C,D)** The effect of AAV2/9-shARAP1 on the expression of ARAP1 and HIF-1α in the mouse kidney tissues was examined by immunofluorescent staining (×400). Bar = 50 μm. Arrows indicate positive staining.

**FIGURE 2:**
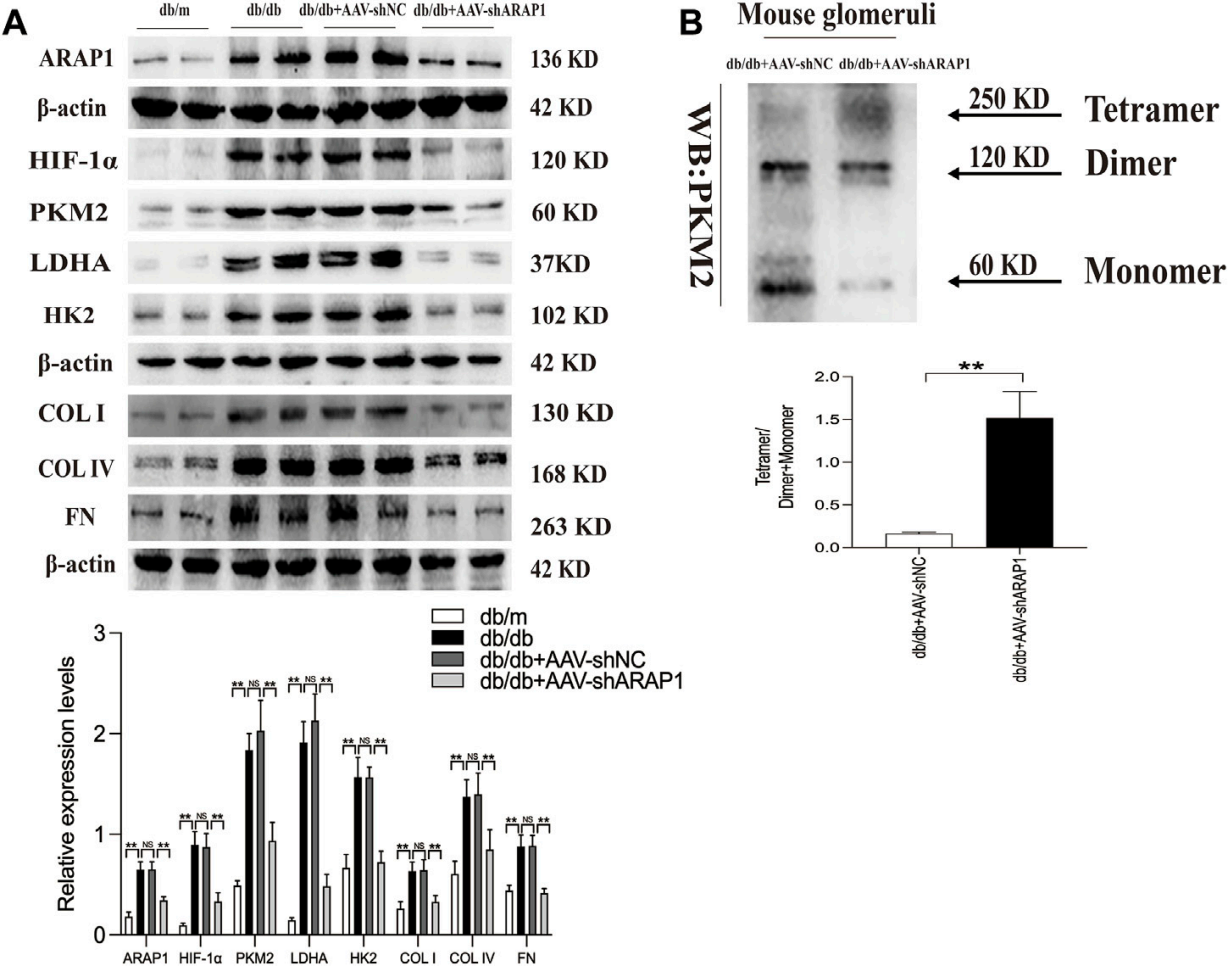
ARAP1 knockdown inhibits dimeric PKM2 expression, HIF-1α accumulation, fibrogenesis and aberrant glycolysis, while partly restore tetrameric PKM2 formation in diabetic mouse **(A)** The effect of AAV2/9-shARAP1 on ARAP1, HIF-1α, PKM2, LDHA, HK2, COL I, COL IV and FN protein expression in the isolated glomeruli lysates of mouse kidney tissues was examined by Western blot analysis. **(B)** Cross-linking detection of PKM2 in the isolated glomeruli lysates of mouse. In all panels, the data are representative of at least three independent experiments. Data are presented as the mean ± SD. *p < 0.05, **p < 0.01, NS, not significant.

The kidney fibrosis program in DKD is associated with an accumulation of HIF-1α and aberrant glycolysis (Li et al., 2020c; Liu et al., 2021). Previous reports have indicated that AAV2/9 can mediate robust ectopic gene expression in renal tissue (Dearborn et al., 2016; Liu et al., 2020). First, MMCs were used to validate the siRNA sequence with the best knockdown effect. ARAP1 mRNA and protein expression was significantly upregulated in high glucose-induced MMCs (Supplementary Figures S3A, B). Mouse siARAP1 (No. c) showed the best knockdown effect (Supplementary Figures S3C, D), and this sequence was chosen for constructing AAV2/9-shARAP1. The sequence is provided in Supplementary Table S5. qRT-PCR results showed that 1 × 10^11^GC/animal of AAV2/9-shARAP1 had the best knockdown effect in db/db mice (Supplementary Figure S3E). The purity of the isolated glomeruli lysates was confirmed by the significant upregulation of Podocin expression, a marker of glomerular podocytes, and the almost complete absence of Cadherin-16 expression, a tubular marker (Supplementary Figure S4). AAV2/9-shARAP1 was injected by tail vein at 13th week (Figure 1). IHC staining results showed that the expressions of ARAP1, HIF-1α, COL I, COL IV and FN were significantly increased in the kidney tissues of db/db mice and were markedly reduced by AAV-shARAP1 (Figure 1B). Immunofluorescence staining validated increased ARAP1 and HIF-1α expression in the kidney tissues of diabetic db/db mice compared with that of db/m mice, and AAV-shARAP1 reduced the expression of ARAP1 and HIF-1α. Moreover, HIF-1α nucleus accumulation was significantly increased in the kidney tissues of db/db mice and were markedly reduced by AAV-shARAP1 (Figures 1C, D). To further confirm our data, the Western blot results showed that the protein expression levels of ARAP1, HIF-1α and glycolysis enzymes, such as PKM2, LDHA, HK2 and the key ECM components COL I, COL IV and FN were significantly increased in isolated glomeruli lysates from diabetic db/db mice compared with that of db/m mice and were markedly reduced by AAV-shARAP1 (Figure 2A). Our cross-linking analysis showed abundant monomeric and dimeric PKM2 expression were markedly reduced by AAV-shARAP1 in diabetic mice glomeruli, while the decreased tetrameric PKM2 expression were partly restored by AAV-shARAP1 (Figure 2B). The overall results indicated that ARAP1 inhibition was highly effective in limiting renal fibrosis and aberrant glycolysis by reducing the PKM2 dimer expression and subsequent HIF-1α nucleus accumulation and activation in diabetic mice.

### 3.2 Knockdown of ARAP1 attenuates renal injury and restores normal renal function in diabetic mice

**FIGURE 3:**
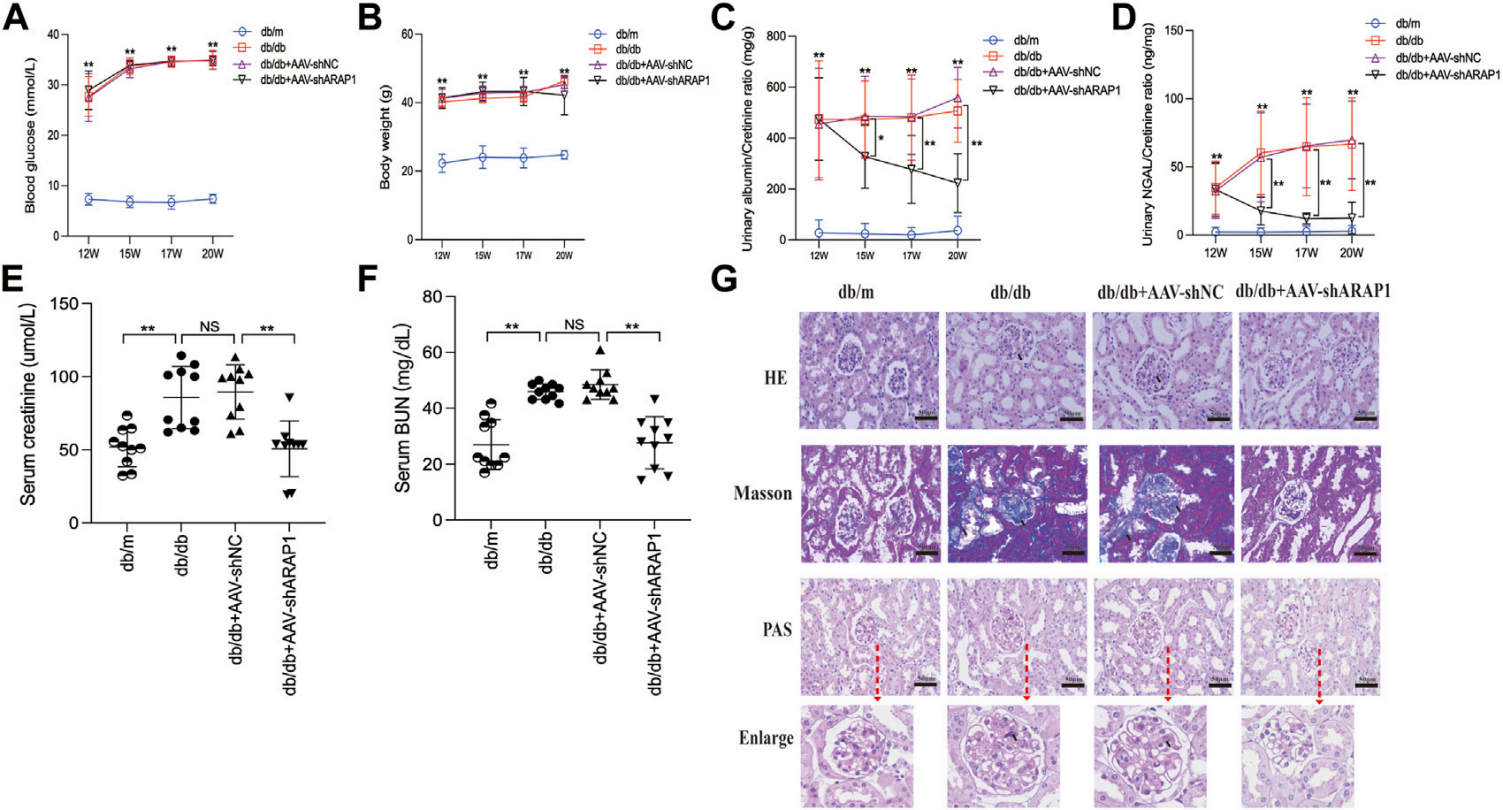
ARAP1 knockdown attenuates renal function and tissue injury in the kidneys of diabetic mouse **(A)** Blood glucose (mmol/L) and **(B)** body weight (g) was measured at the indicated weeks. **(C)** Urinary ACR at various time points (12, 15, 17 and 20 weeks of age). **(D)** Urinary Ngal at various time points (12, 15, 17 and 20 weeks of age). **(E)** Serum creatinine at 20 weeks of age. **(F)** Serum BUN at 20 weeks of age. **(G)** Representative images of HE, PAS and Masson staining (×400). Bar = 50 μm. Arrows indicate positive staining. In all panels, the data are representative of at least three independent experiments. Data are presented as the mean ± SD. *p < 0.05, **p < 0.01, NS, not significant.

The diabetic db/db mice presented with hyperglycemia, which was accompanied by a significant increase in body weight compared with db/m mice. Treatment with AAV-shARAP1 did not affect hyperglycemia or body weight change in diabetic db/db mice (Figures 3A, B). Compared with db/m mice, the UACR of db/db mice was considerably upregulated and was effectively reduced by AAV-shARAP1 treatment. The magnitude of UACR reduction increased as the treatment time extended (Figure 3C). Urinary NGAL, a marker of tubular injury, serum creatinine and BUN were reduced by AAV-shARAP1 treatment in db/db mice (Figures 3D–F). HE, PAS and Masson’s staining showed mesangial matrix expansion and glomerulosclerosis progressed and kidney fibrosis in db/db mice, and AAV-shARAP1 treatment considerably reduced the morphological changes (Figure 3G). Moreover, the GSI score was reduced by AAV-shARAP1 in the kidneys of diabetic mice (PAS staining, Supplementary Figure S5). These data showed the reno-protective effects of ARAP1 gene knockdown in diabetic mice.

### 3.3 Knockdown of ARAP1 inhibits dimeric PKM2 expression HIF-1α expression, pro-fibrotic responses and aberrant glycolysis accompanied by restoring tetrameric PKM2 formation in high glucose-induced human glomerular mesangial cells

**FIGURE 4:**
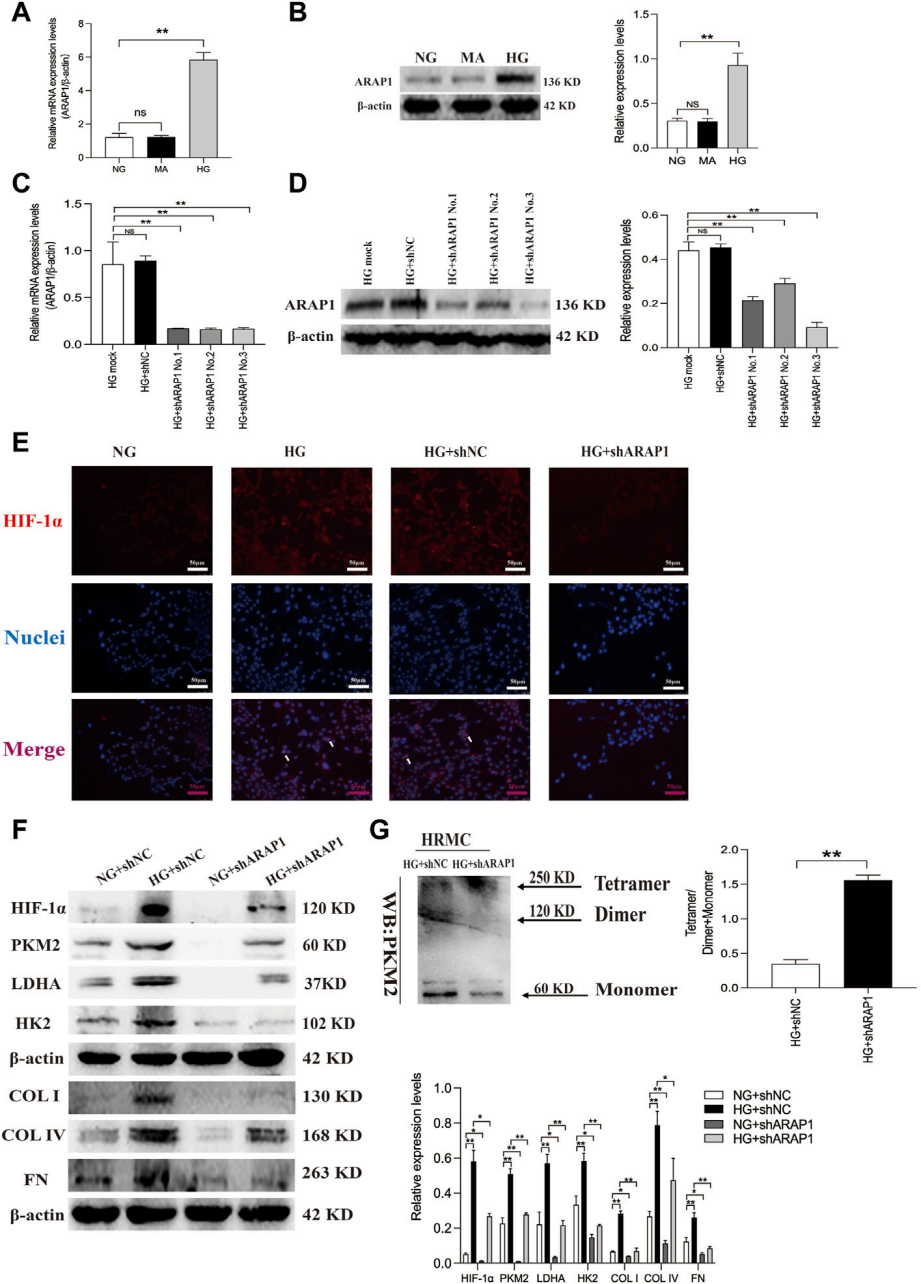
ARAP1 knockdown inhibits high glucose-induced dimeric PKM2 expression, HIF-1α nuclear accumulation, pro-fibrotic responses and aberrant glycolysis, while partly restore tetrameric PKM2 formation in human glomerular mesangial cells. **(A,B)** qRT-PCR and Western blot analysis showed that the mRNA and protein levels of ARAP1 were upregulated in HG group compared with NG group and MA group. **(C,D)** Forty-eight hours after transfection of ARAP1 shRNA (3,000 ng) in the HG group in 6-well plates, the ARAP1 knockdown efficiency was examined by qRT-PCR and Western blot analysis. **(E)** HIF-1α was assessed by immunofluorescence after treatment with high glucose or ARAP1 shRNA (×400). Bar = 50 μm. Arrows indicate positive staining. **(F)** Forty-eight hours after transfection of ARAP1 shRNA in the NG group and HG group, the protein expression of HIF-1α, PKM2, LDHA, HK2, COL I, COL IV and FN was examined by Western blot analysis. **(G)** Cross-linking detection of PKM2 in human glomerular mesangial cells. In all panels, the data are representative of at least three independent experiments. Data are presented as the mean ± SD. *p < 0.05, **p < 0.01, NS, not significant.

The abnormal expression of ARAP1 in high glucose-induced HK-2 cells was reported in our previous study (Yang et al., 2019a; Li et al., 2020a; Li et al., 2020b). We further verified ARAP1 expression in HRMCs and found that ARAP1 was significantly upregulated in HRMCs exposed to the high glucose condition (Figures 4A, B). qRT-PCR and Western blot analysis showed that shARAP1 (No. 3) knocked down ARAP1 expression most effectively in high glucose-induced HRMCs (Figures 4C, D). The shRNA sequence targeting ARAP1 is provided in Supplementary Table S6. Immunofluorescence staining shows the location of increased HIF-1α in nuclei of high glucose-treated HRMCs, while the high glucose-induced HIF-1α nuclear accumulation was reduced by ARAP1 shRNA (Figure 4E). Similar with the observation *in vivo*, high glucose levels induced increased HIF-1α, PKM2, LDHA, HK2, COL I, COL IV and FN were effectively reduced by ARAP1 shRNA treatment (Figure 4F). Our cross-linking analysis showed abundant monomeric and dimeric PKM2 expression were markedly reduced by AAV-shARAP1 in high glucose-induced HRMCs, while the decreased tetrameric PKM2 expression were markedly increased by AAV-shARAP1 (Figure 4G). Overall, the findings indicated that ARAP1 may play a role in promoting dimeric PKM2 expression, HIF-1α accumulation and aberrant glycolysis and driving the pro-fibrotic responses in high glucose-induced HRMCs.

### 3.4 ARAP1 promotes dimeric PKM2 expression accompanied by inhibiting tetrameric PKM2 formation by maintaining persistent EGFR activation through regulating EGFR ubiquitination in DKD

**FIGURE 5:**
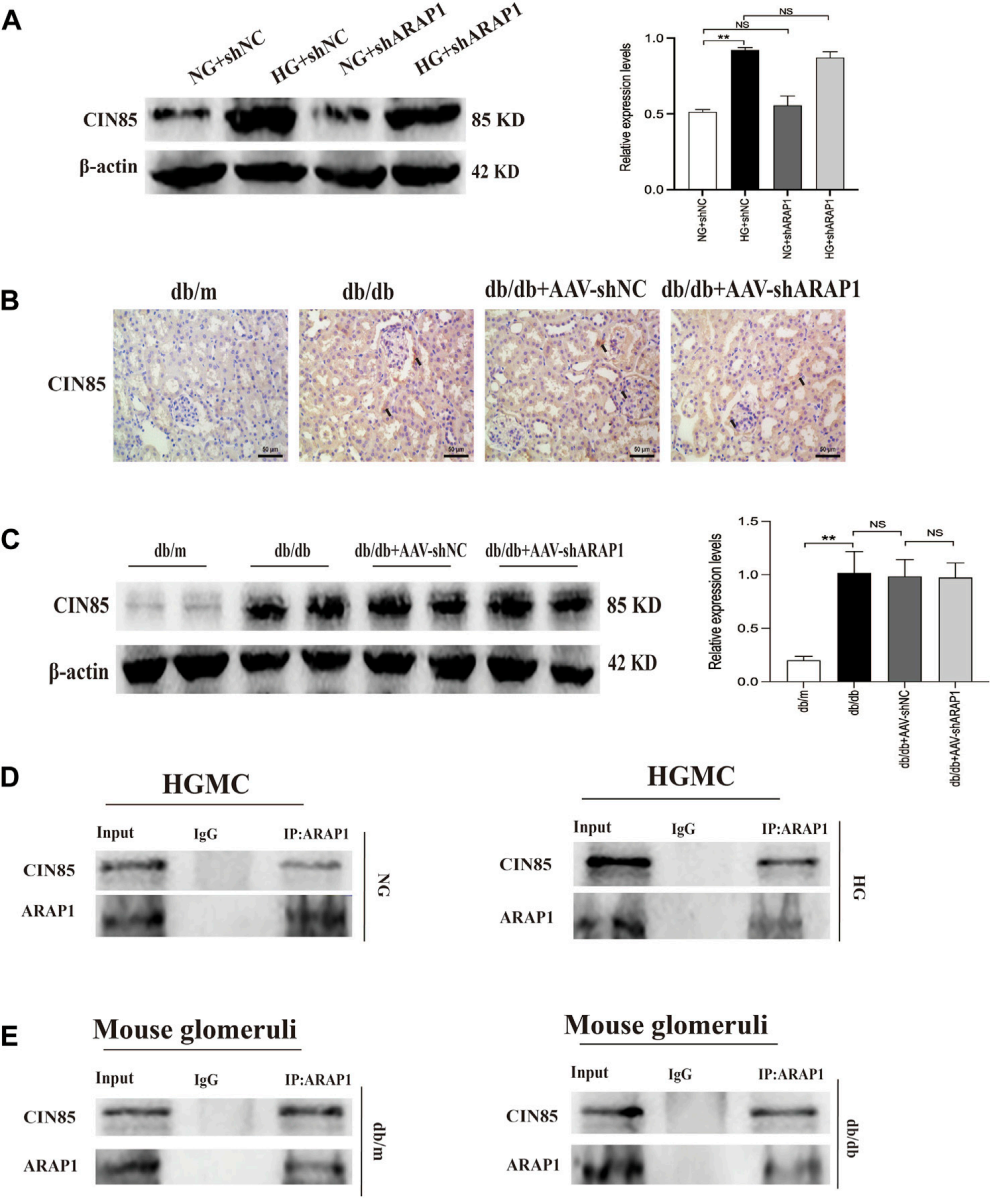
Interaction between ARAP1 and CIN85 **(A)** Forty-eight hours after transfection of ARAP1 shRNA in the NG group and HG group, the protein expression of CIN85 was examined by Western blot analysis. **(B)** IHC detection of CIN85 in kidney tissues from db/m, db/db, db/db + AAV-shNC and db/db + AAV-shARAP1 mice (×400). Bar = 50 μm. Arrows indicate positive staining. **(C)** The effect of AAV2/9-shARAP1 on the protein expression of CIN85 in the isolated glomeruli lysates of mouse kidney tissues was examined by Western blot analysis. **(D,E)** The physical interaction between ARAP1 and CIN85 in human glomerular mesangial cells and mouse glomeruli was detected by Co-IP analysis. IgG was used as a negative control. In all panels, the data are representative of at least three independent experiments. Data are presented as the mean ± SD. *p < 0.05, **p < 0.01, NS, not significant.

We previously reported that ARAP1 interacted with CIN85 and regulates the ubiquitination of EGFR in high glucose-induced human proximal tubular cells (Li et al., 2020b). In the current study, we explored the interaction between ARAP1, CIN85 and EGFR in high glucose-induced HRMCs and diabetic db/db mice. The Western blot results showed that CIN85 was significantly increased in high glucose-induced HRMCs, while ARAP1 shRNA had no effect on CIN85 expression (Figure 5A). IHC staining showed that AAV-shARAP1 had no effect on CIN85 expression in db/db mice (Figure 5B). Likewise, we found that CIN85 protein expression in isolated glomeruli lysates from diabetic db/db mice was not affected by AAV-shARAP1 (Figure 5C). These results collectively suggested that ARAP1 did not regulate the expression of CIN85 in high glucose-induced HRMCs or diabetic db/db mouse glomeruli.

To further confirm that ARAP1 interacts with CIN85, we performed Co-IP assays using protein extracts from HRMCs. The proteins were immunoprecipitated using a mouse anti-ARAP1 antibody or anti-mouse IgG as a negative control, and the precipitates were analyzed by Western blot analysis with a rabbit anti-CIN85 antibody recognizing CIN85. The anti-ARAP1 antibody recognizing ARAP1 successfully precipitated CIN85, but the anti-mouse IgG failed to precipitate CIN85 in normal glucose and high glucose-induced HRMCs (Figure 5D). Consequently, we performed Co-IP assays using protein extracts from isolated glomeruli lysates. The proteins were immunoprecipitated using a rabbit anti-ARAP1 antibody or anti-rabbit IgG as a negative control, and the precipitates were analyzed by Western blot analysis with a rabbit anti-CIN85 antibody recognizing CIN85. The anti-ARAP1 antibody recognizing ARAP1 successfully precipitated CIN85, but the anti-rabbit IgG failed to precipitate CIN85 (Figure 5E). Therefore, our data suggested that ARAP1 could interact with CIN85 in high glucose-induced HRMCs and diabetic db/db mouse glomeruli.

**FIGURE 6:**
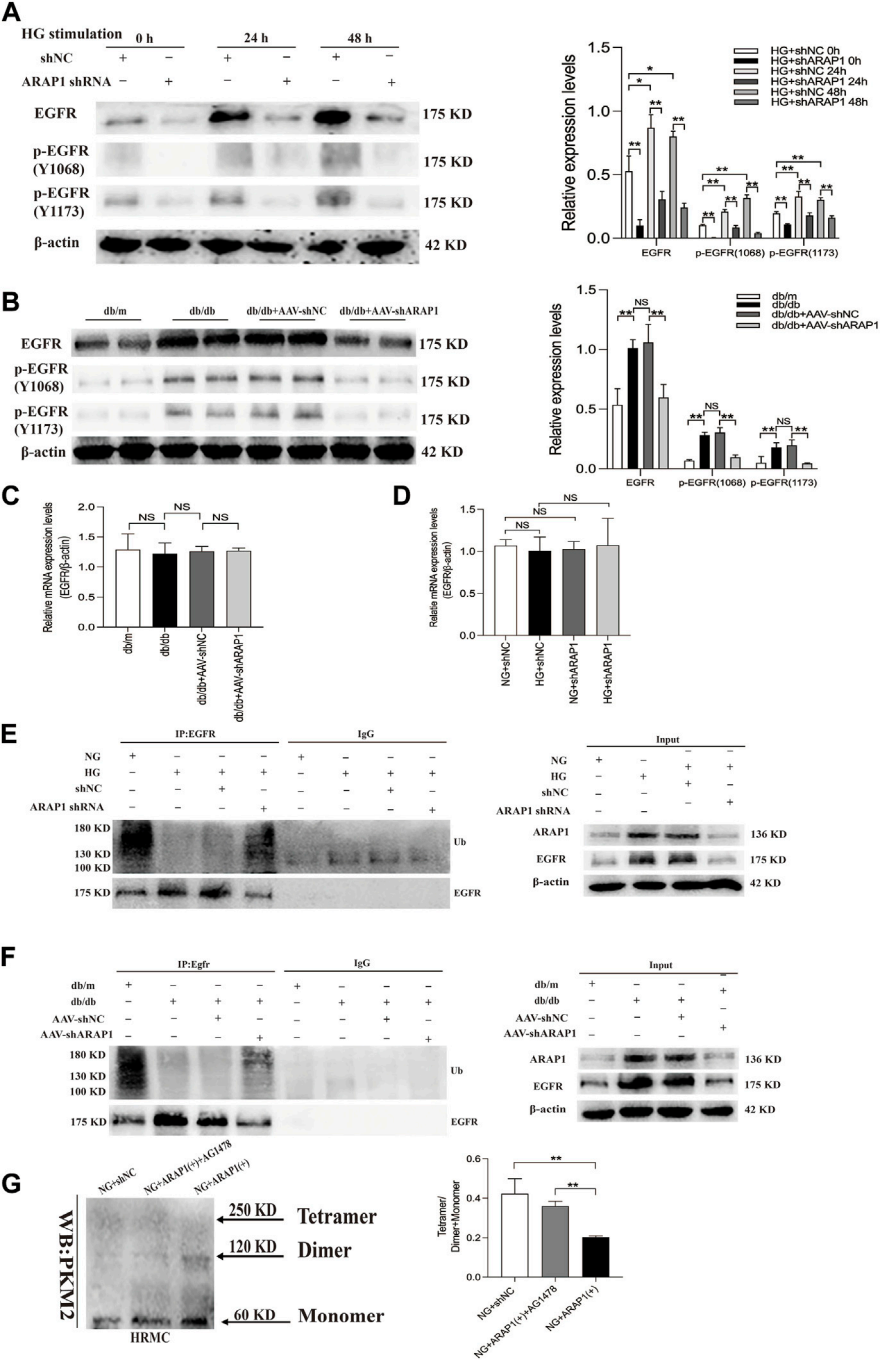
ARAP1 regulates dimeric and tetrameric PKM2 expression by reducing EGFR ubiquitination and maintaining persistent EGFR activation **(A)** Human glomerular mesangial cells were pre-treated with ARAP1 shRNA for 24 h and then divided into the NG group and HG group. The protein expression levels of total EGFR and EGFR phosphorylated at two phosphorylation sites (Y1068 and Y1173) were detected at 24 and 48 h after stimulation by high glucose. HRMCs were pre-treated with ARAP1 shRNA for 24 h and then divided into NG and HG groups. 0 h means that the HRMCs were only transfected with ARAP1 shRNA for 24 h under normal glucose and were not stimulated with high glucose. **(B)** Effect of AAV2/9-shARAP1 on the protein expression of total EGFR, and EGFR phosphorylated at two phosphorylation sites (Y1068 and Y1173) in the isolated glomeruli lysates of mouse kidney tissues was examined by Western blot analysis. **(C)** The mRNA expression of total EGFR in the isolated glomeruli lysates of kidney tissues from the db/m, db/db, db/db + AAV-shNC and db/db + AAV-shARAP1 mice was measured by qRT-PCR. **(D)** Forty-eight hours after transfection of ARAP1 shRNA in the NG group and HG group, the mRNA expression of total EGFR was measured by qRT-PCR. **(E)** ARAP1 knockdown with ARAP1 shRNA increased EGFR ubiquitination in human glomerular mesangial cells cultured with high glucose. **(F)** ARAP1 knockdown with AAV2/9-shARAP1 increased EGFR ubiquitination in the isolated glomeruli lysates of diabetic db/db mouse kidney tissues. **(G)** After transfection of ARAP1 overexpression plasmid accompanied by AG1478 treatment in the NG group, PKM2 monomer, dimer and tetramer in human glomerular mesangial cells were detected by cross-linking assay. In all panels, the data are representative of at least three independent experiments. Data are presented as the mean ± SD. *p < 0.05, **p < 0.01, NS, not significant.

EGFR activation promotes dimer PKM2 expression and nuclear translocation to activate HIF-1α, contributing to abnormal glycolysis and tumor cell proliferation (Yang et al., 2011; Yang et al., 2018; Wang et al., 2020). EGFR activation promotes HIF-1α activation and ECM accumulation in mesangial cells in response to high glucose, which finally resulted in matrix upregulation and glomerular fibrosis (Uttarwar et al., 2011; Li et al., 2015), but the exact regulatory mechanism of ARAP1 on EGFR activation and PKM2/HIF-1ɑ pathway in mesangial cells has not been reported. Therefore, we used HRMCs and glomeruli of diabetic mice to further dissect the regulatory mechanism of ARAP1 on persistent EGFR activation. The Western blot results showed that the expression levels of total EGFR and activation of EGFR as indicated by its phosphorylation at two phosphorylation sites (Y1068 and Y1173) were consistently inhibited after shARAP1 transfection in high glucose-induced HRMCs (Figure 6A). The protein expression levels of total EGFR and EGFR phosphorylated at two phosphorylation sites (Y1068 and Y1173) were upregulated in isolated glomeruli lysates from diabetic db/db mice and were markedly reduced by AAV-shARAP1 (Figure 6B). There was no significant change in total EGFR mRNA expression in cells or mouse glomeruli (Figures 6C, D). Ubiquitination assays revealed that EGFR ubiquitination was significantly reduced in high glucose-induced HRMCs and the glomeruli of db/db mice and was markedly enhanced after ARAP1 knockdown (Figures 6E, F). To clarify the relationship between ARAP1, EGFR and PKM2, we performed a rescue experiment in HRMCs. The transfection efficacy of ARAP1 overexpressed plasmid was verified by Western blot (Supplementary Figure S6G). The results of cross-linking analysis showed that increased abundant monomeric and dimeric PKM2 expression by ARAP1 overexpression plasmids were markedly reduced by AG1478 in normal glucose-induced HRMCs, while the decreased tetrameric PKM2 expression induced by ARAP1 overexpression plasmids were partially restored by AG1478 (Figure 6G). Together, these data demonstrated that the ARAP1 interacts with CIN85 to reduce EGFR ubiquitination and thus stabilized the level of total EGFR proteins to maintain the persistent activation of EGFR and subsequent increased dimeric PKM2 expression as well as decreased tetrameric PKM2 formation in DKD.

### 3.5 YY1 directly promotes ARAP1-AS2 transcriptional activity *in vitro*

**FIGURE 7:**
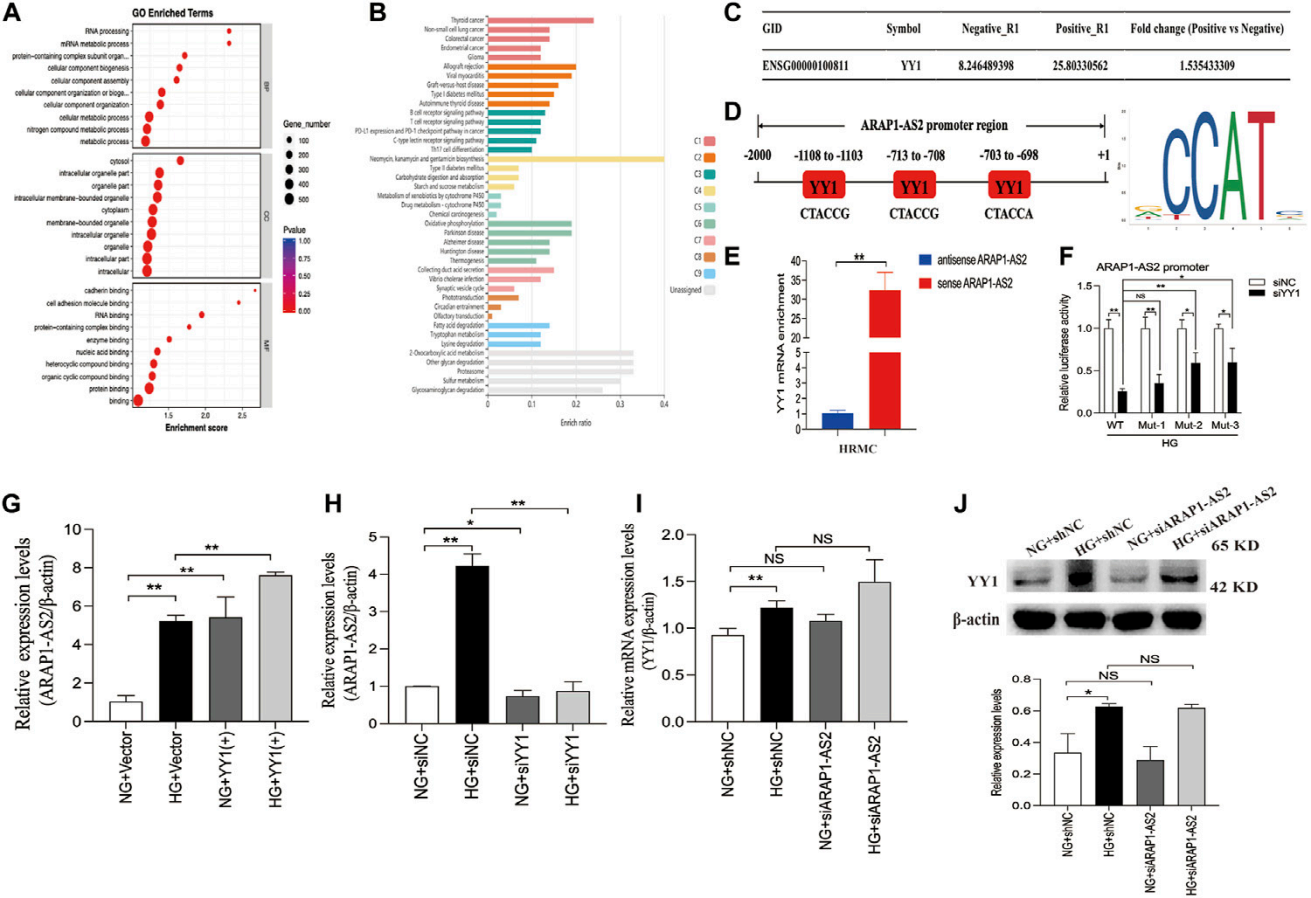
Regulatory mechanism of YY1 on ARAP1-AS2 **(A)** GO analysis of RNA pull-down seq. **(B)** KEGG enrichment analysis of RNA pull-down seq. **(C)** The enrichment of YY1 in the complexes pulled down by ARAP1-AS2. A higher fold change value indicates more enrichment. **(D)** Three YY1 binding sites in the promoter of ARAP1-AS2 were predicted by the JASPAR tool. **(E)** RNA pull-down assays were performed to examine whether ARAP1-AS2 could bind YY1 in HRMCs. YY1 was pulled down by ARAP1-AS2, and the level of YY1 was measured by qRT-PCR. **(F)** Luciferase reporter analysis demonstrated the effect of YY1 silencing on the luciferase activity of the ARAP1-AS2 promoter reporter in indicated experimental groups. **(G,H)** Forty-eight hours after transfection of the YY1 overexpression plasmid or YY1 siRNA in the NG group and HG group, the expression level of ARAP1-AS2 was measured by qRT-PCR. **(I)** Forty-eight hours after transfection of the ARAP1-AS2 siRNA in the NG group and HG group, the mRNA expression level of YY1were measured by qRT-PCR. **(J)** Forty-eight hours after transfection of the ARAP1-AS2 siRNA in the NG group and HG group, the protein expression level of YY1 was examined by Western blot analysis. In all panels, the data are representative of at least three independent experiments. Data are presented as the mean ± SD. *p < 0.05, **p < 0.01, NS, not significant.

YY1 is known to interact with LncRNAs and regulate the transcriptional activity of LncRNAs (An and Ding, 2021). Results of the RNA pulldown-seq of ARAP1-AS2 in HK-2 cells were reported in our previous study (Li et al., 2020b). To explore the potential biological function of the genes enriched in ARAP1-AS2 pulldown, GO and KEGG pathway enrichment analyses were conducted. GO analyses revealed these genes to be mainly enriched in “metabolic process” (BP), “RNA and protein binding” (MF) and “membrane-bounded organelle” (CC) (Figure 7A). According to KEGG analysis, the enriched genes were closely related to diabetes mellitus and the ubiquitin-mediated proteolysis pathway (Figure 7B; Supplementary Table S7). The complexes pulled down were enriched in YY1 (Figure 7C). We identified 3 YY1 binding sites in the ARAP1-AS2 promoter using the JASPAR tool (Figure 7D). RNA pull-down assays showed that YY1 was pulled down only by sense ARAP1-AS2 in HRMCs (Figure 7E). Luciferase reporter analysis demonstrated that in HRMCs stimulated with high glucose, siYY1 reduced the activity of the ARAP1-AS2 promoter, and mutations at sites 2 and 3, but not site 1, partly restored the promoter activity (Figure 7F). These data proved that YY1 targeted the ARAP1 promoter at sites 2 and 3. We further verified that ARAP1-AS2 and YY1 were significantly upregulated in high glucose-induced HRMCs (Supplementary Figures S6A, C, D). We transfected the siRNAs targeting ARAP1-AS2 and YY1, YY1 overexpression plasmids and verified their activity (Supplementary Figures S6B, E, F). The siRNA sequence targeting ARAP1-AS2 and YY1 is described in Supplementary Table S5. The results of qRT-PCR revealed that the levels of ARAP1-AS2 were significantly upregulated by YY1 overexpression plasmids, while the expression of ARAP1-AS2 was decreased by YY1 siRNAs (Figures 7G, H). There was no change in the mRNA and protein expression of YY1 after ARAP1-AS2 knockdown (Figures 7I, J). These results indicate that YY1 directly and positively regulates ARAP1-AS2 transcriptional activity in high glucose-induced HRMCs.

### 3.6 YY1 regulate EGFR activation, HIF-1α accumulation and aberrant glycolysis and ECM accumulation in human glomerular mesangial cells, possibly through the ARAP1-AS2/ARAP1 axis

**FIGURE 8:**
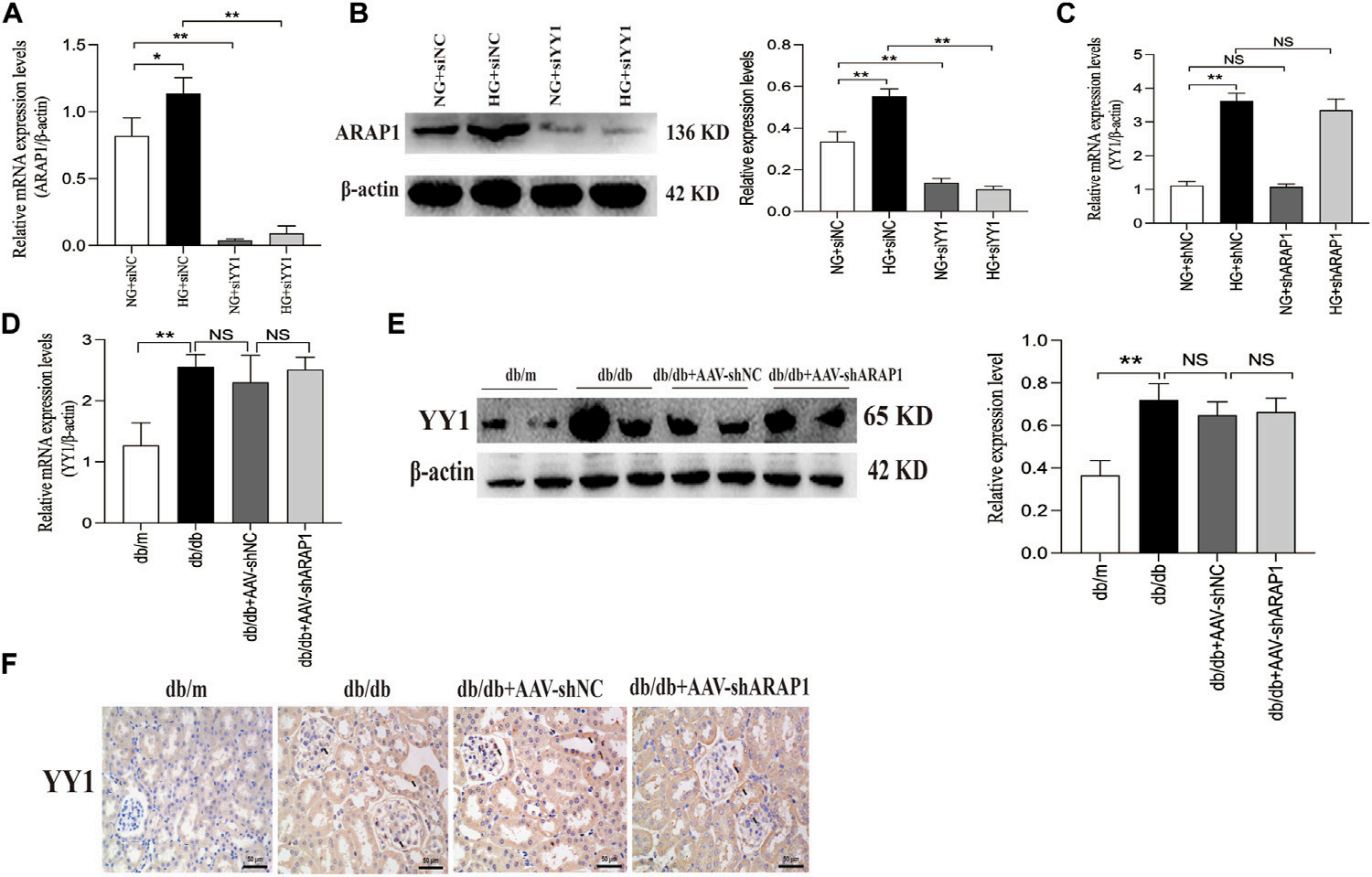
Effect of YY1 on ARAP1 **(A)** Forty-eight hours after transfection of the YY1 siRNA in the NG group and HG group, the mRNA expression level of ARAP1were measured by qRT-PCR. **(B)** Forty-eight hours after transfection of the YY1 siRNA in the NG group and HG group, the protein expression level of ARAP1 was examined by Western blot analysis. **(C)** Forty-eight hours after transfection of the ARAP1 shRNA in the NG group and HG group, the mRNA expression level of YY1 were measured by qRT-PCR. **(D)** The mRNA expression of YY1 in the isolated glomeruli lysates of kidney tissues from the db/m, db/db, db/db + AAV-shNC and db/db + AAV-shARAP1 mice was measured by qRT-PCR. **(E)** The protein expression of YY1 in the isolated glomeruli lysates of kidney tissues from the db/m, db/db, db/db + AAV-shNC and db/db + AAV-shARAP1 mice was measured by Western blot analysis. **(F)** IHC detection of YY1 in kidney tissues from db/m, db/db, db/db + AAV-shNC and db/db + AAV-shARAP1 mice (×400). Bar = 50 μm. Arrows indicate positive staining.

We further verified that the mRNA and protein expression of ARAP1 were significantly downregulated after YY1 knockdown (Figures 8A, B). There was no change in the mRNA expression of YY1 after ARAP1 knockdown (Figure 8C). Similar with the *in-vitro* results, we found that the increased mRNA and protein expressions of YY1 in isolated glomeruli lysates from diabetic db/db mice was not affected by AAV-shARAP1 (Figures 8D, E). Likewise, IHC staining showed that AAV-shARAP1 had no effect on YY1expression in db/db mice (Figure 8F).

**FIGURE 9:**
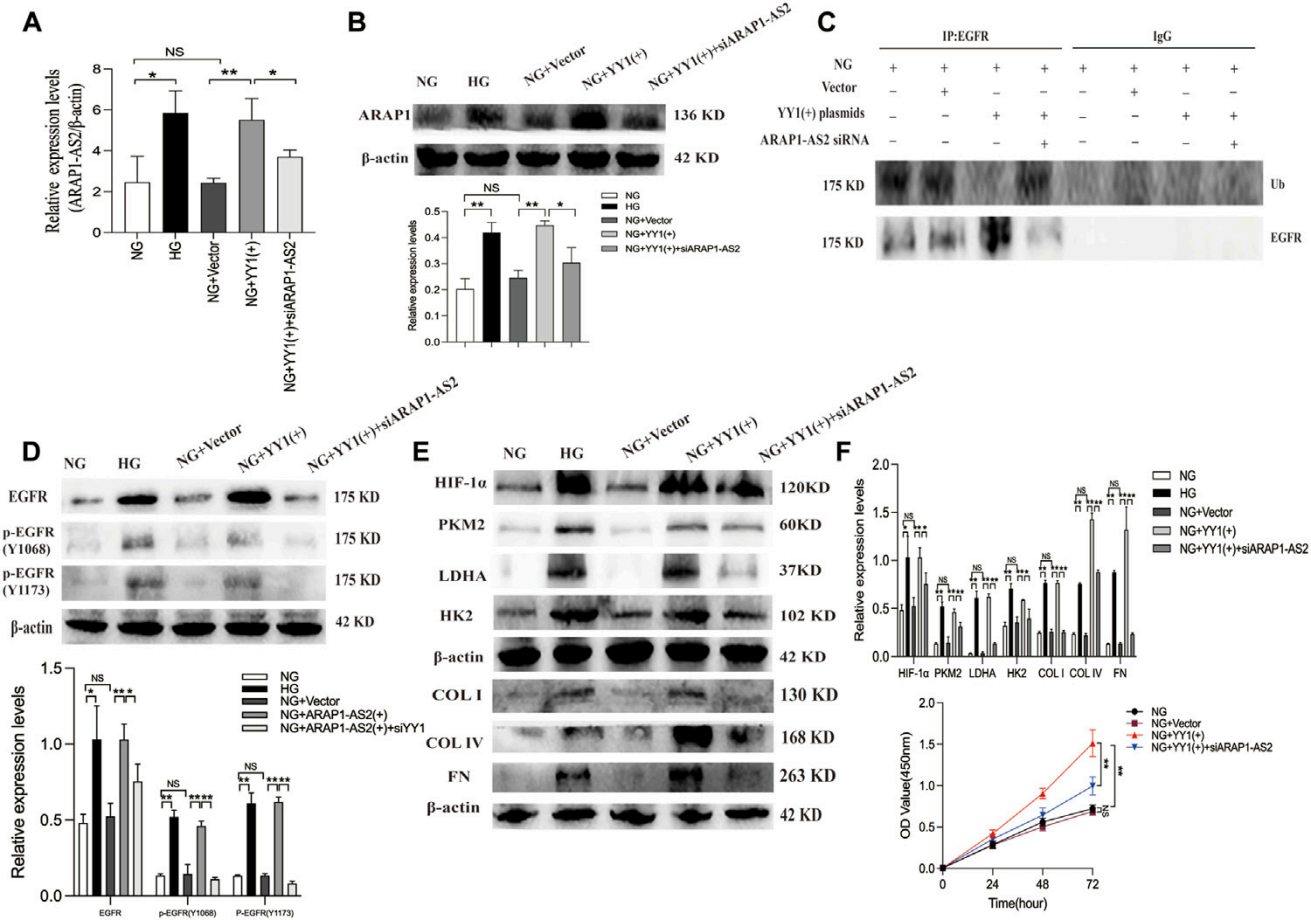
YY1 regulate EGFR ubiquitination and activation, HIF-1α accumulation and aberrant glycolysis and ECM accumulation in human glomerular mesangial cells through the ARAP1-AS2/ARAP1 axis. **(A)** Forty-eight hours after co-transfection of the YY1 overexpression plasmid and ARAP1-AS2 siRNA in the NG group, the expression level of ARAP1-AS2 was examined by qRT-PCR. **(B)** Forty-eight hours after co-transfection of the YY1 overexpression plasmid and ARAP1-AS2 siRNA in the NG group, the protein expression levels of ARAP1 were examined by Western blot analysis. **(C)** Forty-eight hours after co-transfection of the YY1 overexpression plasmid and ARAP1-AS2 siRNA in the NG group, EGFR ubiquitination were examined. **(D)** Forty-eight hours after co-transfection of the YY1 overexpression plasmid and ARAP1-AS2 siRNA in the NG group, the protein expression levels of total EGFR, and EGFR phosphorylated at two phosphorylation sites (Y1068 and Y1173) were examined by Western blot analysis. **(E)** Forty-eight hours after co-transfection of the YY1 overexpression plasmid and ARAP1-AS2 siRNA in the NG group, the protein expression levels of HIF-1α, PKM2, LDHA, HK2, COL I, COL IV and FN were examined by Western blot analysis. **(F)** After co-transfection of the YY1 overexpression plasmid and ARAP1-AS2 siRNA, cell proliferation was evaluated by CCK-8 assay at 0, 24, 48 and 72 h. In all panels, the data are representative of at least three independent experiments. Data are presented as the mean ± SD. *p < 0.05, **p < 0.01, NS, not significant.

To clarify the relationship between YY1, ARAP1-AS2, ARAP1, EGFR activation, HIF-1α accumulation and ECM accumulation, we performed a rescue experiment in HRMCs. The results of qRT-PCR showed that the effect of YY1 overexpression on increasing the levels of ARAP1-AS2 in the NG group was inhibited by siARAP1-AS2 (Figure 9A). Western blot analysis showed that the effect of YY1 overexpression on increasing the levels of ARAP1, total EGFR and EGFR phosphorylated at two phosphorylation sites (Y1068 and Y1173), HIF-1α,PKM2, LDHA, HK2 and ECM components COL I, COL IV and FN in the NG group was inhibited by siARAP1-AS2 (Figures 9B, D, E). Ubiquitination assays revealed that the effect of YY1 overexpression on reducing EGFR ubiquitination was significantly restored by siARAP1-AS2 in high glucose-induced HRMCs (Figure 9C). Moreover, the CCK-8 assay revealed that the effect of YY1 overexpression on promoting cell proliferation in the NG group was blocked by siARAP1-AS2 (Figure 9F). These results demonstrate that YY1 may have regulated EGFR ubiquitination and activation, HIF-1α accumulation and aberrant glycolysis and ECM accumulation in human glomerular mesangial cells through the ARAP1-AS2/ARAP1 axis in human glomerular mesangial cells.

## 4 Discussion

Recent studies have shown that HIF-1α is activated to induce aberrant transcription of genes encoding glycolytic enzymes in the pathogenesis of DKD (Isoe et al., 2010; Liu et al., 2021). PKM2 can be aggregated into tetramer and dimer forms. Dimeric PKM2 acts as a key protein kinase in aberrant glycolysis by promoting the accumulation of HIF-1α, while tetrameric PKM2 functions as a pyruvate kinase in oxidative phosphorylation (Luo et al., 2011; Sizemore et al., 2018). The transformation between them plays an important role in energy supply of tumor cells, epithelial-mesenchymal transition (EMT), invasion and metastasis and cell proliferation (Zhang et al., 2019a). It has been proved that the PKM2 tetramer form decreases, the dimer form increases and enters the nucleus to activate HIF-1α in DKD (Liu et al., 2021). In this study, we first verified that ARAP1 knockdown can alleviate ECM accumulation and renal dysfunction and glomerulosclerosis in diabetic db/db mice as well as in GMCs exposed to high glucose through reducing aberrant glycolysis *via* decreasing HIF-1α nuclear accumulation, possibly associated with reduced expression and nucleus translocation of dimer PKM2 accompanied by restoring tetramer formation led by breaking the EGFR persistent transactivation. We provide the first evidence for YY1 regulate EGFR activation, dimer PKM2 accumulation, HIF-1α activation and aberrant glycolysis and ECM accumulation in DKD through the ARAP1-AS2/ARAP1 axis.

One of the characteristics of DKD is ECM accumulation, which eventually leads to glomerular sclerosis and fibrosis (Doi et al., 2018). Studies have demonstrated that HIF-1α promotes aberrant glycolysis, which finally leading to ECM accumulation and renal fibrosis in mouse models of chronic/hypoxic renal injury models (Cai et al., 2020; Wei et al., 2022). Our diabetic db/db mice showed the pro-fibrotic activities in the kidney accompanied by kidney dysfunction and increased proteinuria and kidney injury in this study, indicating that our diabetic model presented with progressive DKD. Our group has reported excessive ECM accumulation and kidney fibrosis in diabetic db/db mice (Zhang et al., 2019b; Ma et al., 2022). Our cross-linking experiment first demonstrated that ARAP1 inhibition reduced PKM2 dimer expression and partially restored tetramer formation in high glucose-induced HRMCs and db/db mouse glomeruli. The HIF-1α activation, aberrant glycolytic genes expression and pro-fibrotic activities, such as the increased collagen accumulation in kidney glomeruli, kidney dysfunction and kidney injury, were significantly prevented after administration of AAV2/9-shARAP1in the diabetic mice.

Mesangial cells can cause glomerular injury through excessive cell proliferation and ECM accumulation (Wang et al., 2018b) and maintain the glomerular capillary structural architecture and mesangial matrix homeostasis (Abboud, 2012). Excessive accumulation of ECM in the glomerulus leads to glomerulosclerosis and contributes to the initiation and progression of DKD. In the present study, we found that ARAP1 was markedly increased in db/db mouse glomeruli and that ARAP1 and its natural antisense lncRNA, ARAP1-AS2, were significantly upregulated in high glucose stimulated-HRMCs, which increased the accumulation of PKM2 dimer accompanied by reducing tetramer formation, activation of HIF-1α and expression of aberrant glycolytic and ECM genes. To date, the regulatory mechanism of ARAP1-AS2 in DKD has been reported only in HK-2 cells by our study team (Li et al., 2020a; Li et al., 2020b), while the potential involvement of other renal cell populations and its effect in animal models remained unknown. This study also provides a new finding that ARAP1-AS2 is a novel target of YY1 and positively regulated by YY1 in DKD. Luciferase reporter analysis and rescue experiments demonstrated that YY1 targets ARAP1-AS2 promoter and promotes EGFR activation, HIF-1α accumulation and aberrant glycolysis and ECM accumulation through the ARAP1-AS2/ARAP1 axis in high glucose stimulated-HRMCs. All these findings further confirmed the important role of ARAP1-AS2/ARAP1 in the pathogenesis of DKD as reported by our group (Yang et al., 2019a; Li et al., 2020a; Li et al., 2020b), but there may also be limitations to this study, as our current data cannot exclude the role of lncRNA-ARAP1-AS2/ARAP1 in other cell types, such as podocytes, in DKD.

**FIGURE 10:**
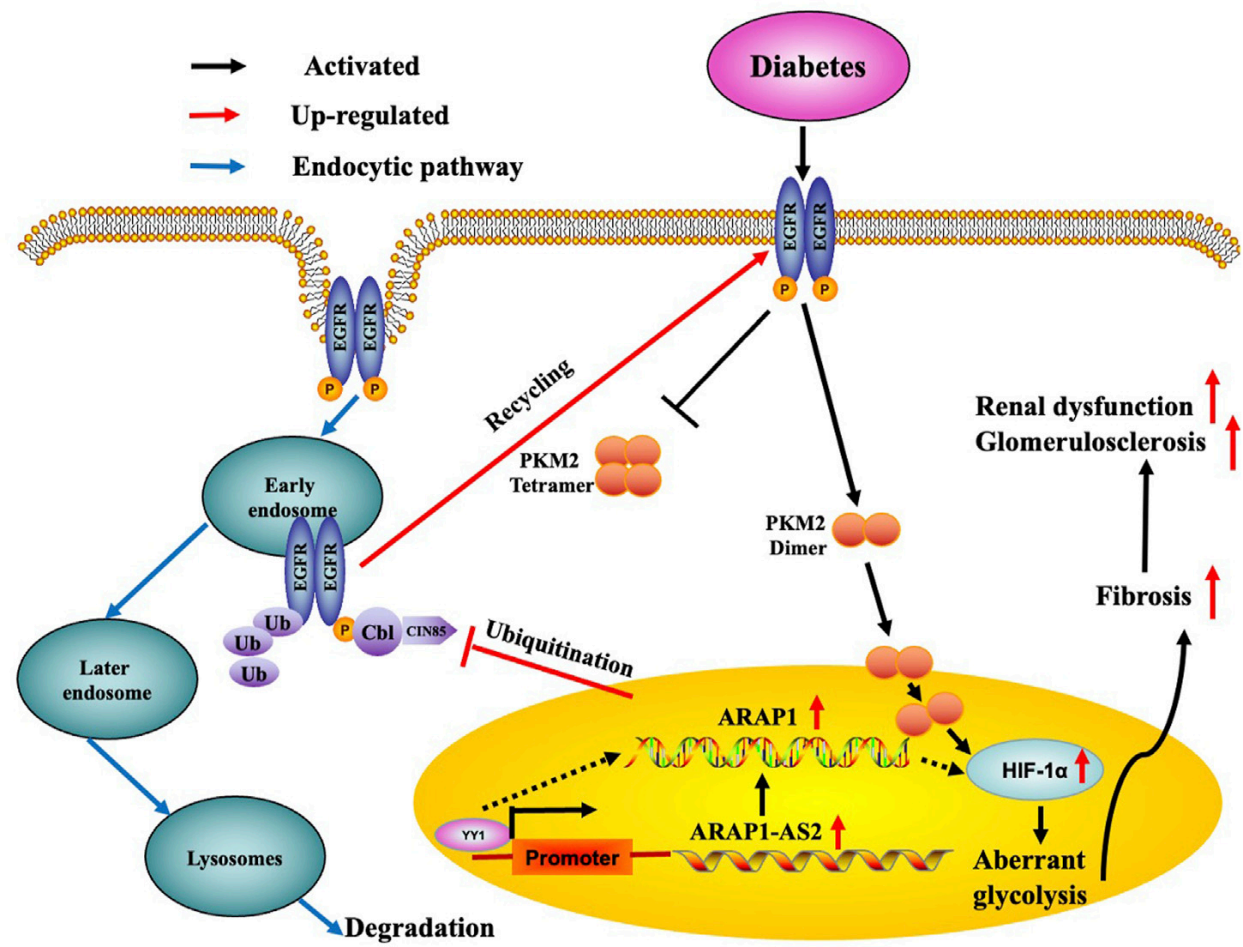
Schematic representation of the proposed model: possible regulatory mechanism of YY1, ARAP1-AS2, and ARAP1 on HIF-1α accumulation and aberrant glycolysis in DKD. Increased YY1 expression in a high-glucose environment can upregulate ARAP1-AS2 expression by targeting its promoter and then indirectly upregulate ARAP1 expression. Subsequently, ARAP1 binds to CIN85 and reduces the ubiquitination of EGFR, thus stabilizing total EGFR protein levels to support the persistent activation of EGFR in DKD. The persistent activation of EGFR then promotes PKM2 dimer expression and nucleus translocation to activate HIF-1α, accompanied by reducing tetramer formation, which finally leads to aberrant glycolysis and aggravates ECM accumulation and glomerulosclerosis and fibrosis in human glomerular mesangial cells exposed to high glucose and diabetic db/db mice.

EGFR is widely expressed in the glomeruli (Chen et al., 2015) and HIF-1α has been clearly demonstrated to be a downstream target of EGFR (Wang et al., 2018c; Wang et al., 2022). EGFR activation has been reported to participate in the activation of HIF-1α by promoting dimer PKM2 expression and enter nucleus, eventually leading to ECM accumulation and renal fibrosis in DKD (Yang et al., 2011; Overstreet et al., 2017). We previously reported that ARAP1 could maintain persistent EGFR activation by reducing the ubiquitination of EGFR through interacting with CIN85 and that ARAP1-AS2 directly interacted with ARAP1 and promoted ARAP1 expression and then regulated CIN85 indirectly in HK-2 cells (Li et al., 2020b); however, the mechanism of persistent EGFR activation in other renal cell populations and animal models of DKD is still unknown. Currently, we focus on the mechanism of EGFR activation in HRMCs and diabetic mice. We found that the interaction between ARAP1 and CIN85 exist in high glucose-stimulated HRMCs and diabetic db/db mouse glomeruli, while ARAP1 knockdown had no effect on CIN85 expression. Our results suggest that ARAP1 knockdown could maintain the decreased levels of total EGFR and the sustained inhibition of EGFR activation. The reduced ubiquitination level of EGFR was significantly reversed after ARAP1 knockdown in high glucose-stimulated HRMCs and db/db mouse glomeruli. However, qRT-PCR results showed that ARAP1 has no effect on the transcription of EGFR. Cross-linking rescue experiment results showed that increased dimeric PKM2 expression induced by ARAP1 overexpression plasmids were markedly reduced by AG1478 in normal glucose-induced HRMCs, while the decreased tetrameric PKM2 expression were partially restored by AG1478. All these findings confirmed ARAP1 can promote dimeric PKM2 expression as well as inhibit tetrameric PKM2 formation by maintaining persistent EGFR activation in high glucose-stimulated HRMCs and diabetic db/db mouse glomeruli. Based on the above results, we indicated that YY1 targets the promoter of ARAP1-AS2 and promotes ARAP1-AS2 and its sense target gene ARAP1 expression. YY1 promotes HIF-1α accumulation and aberrant glycolysis through the ARAP1-AS2/ARAP1 axis, associated with maintaining the EGFR persistent transactivation and increased PKM2 dimer expression and nucleus translocation accompanied by reducing tetramer formation, which finally leads to ECM accumulation and glomerulosclerosis and fibrosis in high glucose-induced human glomerular mesangial cells and diabetic db/db mice (Figure 10).

In summary, our results revealed that ARAP1-AS2/ARAP1 play a role in human glomerular mesangial cells and diabetic db/db mice glomeruli. YY1-induced upregulation of ARAP1-AS2 and ARAP1 promotes kidney fibrosis by HIF-1α accumulation and aberrant glycolysis through promoting EGFR persistent activation and PKM2 dimer expression and nucleus translocation accompanied by reducing tetramer formation. The novel mechanism of YY1, ARAP1-AS2, and ARAP1 in regulating aberrant glycolysis and fibrosis by EGFR/PKM2/HIF-1α pathway will further enhance our understanding of lncRNA functions in DKD and may suggest a potential therapeutic strategy for DKD.

## Data Availability

The original contributions presented in the study are included in the article/Supplementary Material, further inquiries can be directed to the corresponding author.
