# Supplementary material for: YY1-induced upregulation of LncRNA-ARAP1-AS2 and ARAP1 promotes diabetic kidney fibrosis via aberrant glycolysis associated with EGFR/PKM2/HIF-1α pathway
Source: Front Pharmacol. 2023 Feb 15;14:1069348. doi: 10.3389/fphar.2023.1069348 (PMC9974832; doi:10.3389/fphar.2023.1069348)
Supplement: Supplementary file 1 [file DataSheet1.docx]

**Supplementary Table S1.** **The viral vector construction framework of AAV2/9-shARAP1**

| **Gene** | **viral vector construction framework** |
| --- | --- |
| ARAP1 | 5′-CcggCCGGGACTTTGAACGTCTTTTCTCGAGAAGACGTTCAAAGTCCCGGTTTTTTTT-3′ |

**Supplementary Table S2.** **The primary antibody dilution ratios of IHC**

| **Antibody** | **Dilution ratio** | **Source** |
| --- | --- | --- |
| anti-ARAP1  anti-YY1 | 1:50  1:100 | sc-393138, Santa Cruz Biotechnology, USA  ab109228, Abcam, USA |
| anti-CIN85 | 1:50 | ab151574, Abcam, USA |
| anti-HIF-1α | 1:200 | NB100-123, Novus, USA |
| anti-Collagen I | 1:500 | 14695-1-AP, Proteintech Co Ltd, USA |
| anti-Collagen IV | 1:400 | ab6586, Abcam, USA |
| anti-Fibronectin | 1:50 | ab2413, Abcam, USA |

**Supplementary Table S3.** **Primer sequences of qRT-PCR**

| **Gene** | **Primer sequence** |
| --- | --- |
| ARAP1-AS2  （human） | Reverse: GTGGAATGAGGAGCCGAATGAAGG  Forward: GTGCCTAACCTGTCAGCCAATGG |
| ARAP1  （mouse） | Reverse: AGGAACAAAGCGCTTAGAGTAG  Forward: TATCTATCAGAAGCGATGGGTG |
| ARAP1  （human） | Reverse: CAGCCAGCCAGCCTTGATGAC  Forward: CATACCAGCAGCCTGAGCTTGTC |
| YY1  （mouse） | Reverse: TTCTGCTCCCACTTCTTATTCC  Forward: GACGACTACATAGAGCAGACG |
| YY1  （human） | Reverse: GAGGCATATTTATTCCCAATCACAC  Forward: TTGCTCAGTCAACTAACCTGAAATC |
| EGFR  （mouse） | Reverse: GAATGCGTCATCTATGTTGTCC  Forward: TGAGTTCTCTGAGTGCAACTAG |
| EGFR  （human） | Reverse: GAATTCGATGATCAACTCACGG  Forward: ACCCATATGTACCATCGATGTC |
| ACTB  （mouse） | Reverse: CACAGCTTCTCTTTGATGTCAC  Forward: CTACCTCATGAAGATCCTGACC |
| ACTB  （human） | Reverse: CGAGCTCTGAGCACTGGAGA  Forward: TGGCGTGTAAAGTCACCACC |

**Supplementary Table S4. The specific antibody dilution ratios of Western blot**

| **Antibody** | **Dilution ratio** | **Source** |
| --- | --- | --- |
| anti-ARAP1  anti-YY1 | 1:100  1:1000 | sc-393138, Santa Cruz Biotechnology, USA  ab109228, Abcam, USA |
| anti-EGFR | 1:1000 | #54359, CST, USA |
| anti-p-EGFR（Y1173） | 1:1000 | #4407, CST, USA |
| anti-p-EGFR（Y1068） | 1:1000 | #8543, CST, USA |
| anti-HIF-1α | 1:1000 | NB100-123, Novus, USA |
| anti-PKM2 | 1:1000 | 15822-1-AP, Proteintech, USA |
| anti-LDHA | 1:2000 | 19987-1-AP, Proteintech, USA |
| anti-HK2 | 1:2000 | 22029-1-AP, Proteintech, USA |
| anti-COLI | 1:1000 | 14695-1-AP, Proteintech, USA |
| anti-COL IV | 1:1000 | ab6586, Abcam, USA |
| anti-FN | 1:1000 | ab2413, Abcam, USA |

**Supplementary Table S5. The siRNA sequences**

| **Gene** | **siRNA sequence** |
| --- | --- |
| Human ARAP1-AS2 | 5′-GCAAAGUCCUUAGCACUUATT-3′ |
| Human YY1 | 5′-GACGACUACAUUGAACAATT-3′ |
| Mouse ARAP1 | 5′-CCGGGACUUUGAACGUCUUTT-3′ |

**Supplementary Table S6. The shRNA sequence targeting human ARAP1**

| **Gene** | **shRNA sequence** |
| --- | --- |
| ARAP1 | 5′-GCTTCCACGATCGCTACTTCATTCAAGAGATGAAGTAGCGATCGTGGAAGCTTTTTT-3′ |

**Supplementary Table S7. KEGG pathway enrichment analysis**

| pathway | description | count in gene set | false discovery rate |
| --- | --- | --- | --- |
| hsa04141 | Protein processing in endoplasmic reticulum | 19 of 161 | 0.00045 |
| hsa05169 | Epstein-Barr virus infection | 20 of 194 | 0.00079 |
| hsa03010 | Ribosome | 16 of 130 | 0.00079 |
| hsa05166 | HTLV-I infection | 21 of 250 | 0.0040 |
| hsa05203 | Viral carcinogenesis | 16 of 183 | 0.0155 |
| hsa03040 | Spliceosome | 13 of 130 | 0.0155 |
| hsa04120 | Ubiquitin mediated proteolysis | 13 of 134 | 0.0163 |
| hsa00230 | Purine metabolism | 15 of 173 | 0.0163 |
| hsa05131 | Shigellosis | 8 of 63 | 0.0308 |
| hsa01100 | Metabolic pathways | 58 of1250 | 0.0308 |
| hsa05012 | Parkinson's disease | 12 of 142 | 0.0469 |

**
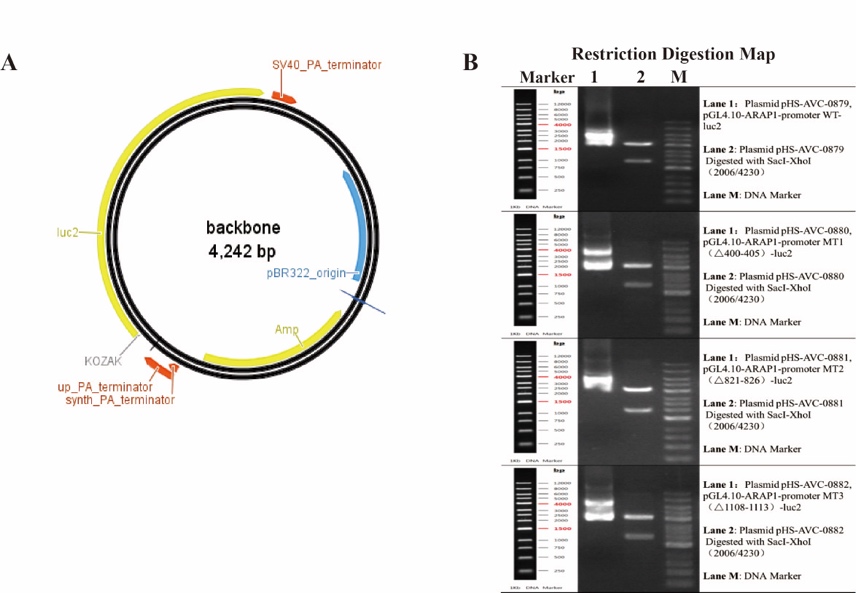
**

**Figure S1. ARAP1-AS2-promoter-luc plasmid backbone**


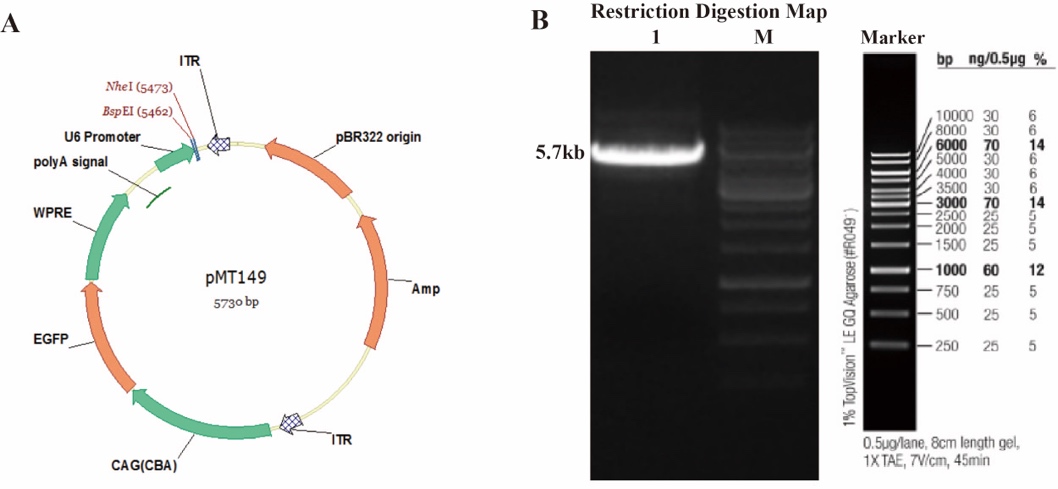


**Figure S2. Adeno-associated virus vector backbone**

**
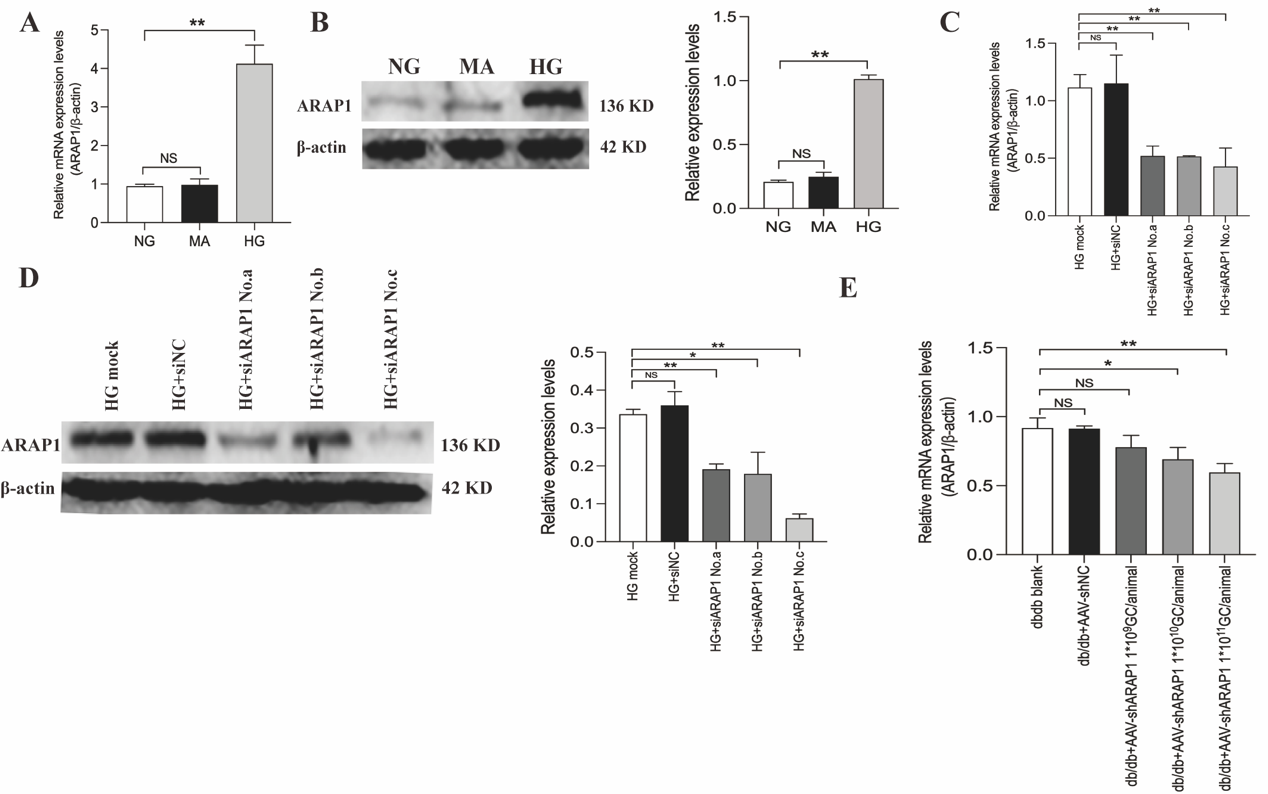
**

**Figure S3. Mouse mesangial cells（MMCs） were used to validate the best knockdown effect ARAP1 siRNA sequence.**

(A) qRT-PCR results showed that ARAP1 mRNA expression was significantly up-regulated in high glucose-induced MMCs. (B) Western blot analysis results showed that ARAP1 protein expression was significantly up-regulated in high glucose-induced MMCs. (C) qRT-PCR results showed that siARAP1 (No. c) had the best knockdown effect on ARAP1 mRNA expression in high glucose-induced MMCs. (D) Western blot analysis results showed that siARAP1 (No. c) had the best knockdown effect on ARAP1 protein expression in high glucose induced MMCs. (E) qRT-PCR result showed that 1×10^11^GC/animal of AAV2/9-shARAP1 injection through mouse tail vein had the best knockdown effect on ARAP1 mRNA expression in glomeruli of db/db mice and we chose 1×10^11^GC/animal of AAV-shARAP1 for this study. In all panels, the data are representative of three independent experiments. Data is presented as the mean±SD. **P*<0.05, ***P*<0.01, NS, not significant.


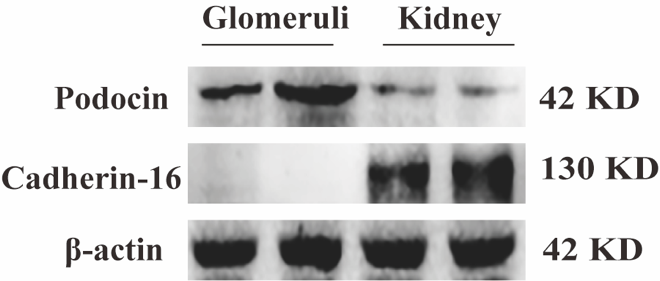


**Figure S4.** (A) The expression of Podocin, a glomerular podocyte marker, and the expression of Cadherin-16, a tubular marker in the isolated glomeruli and the total kidney lysate.


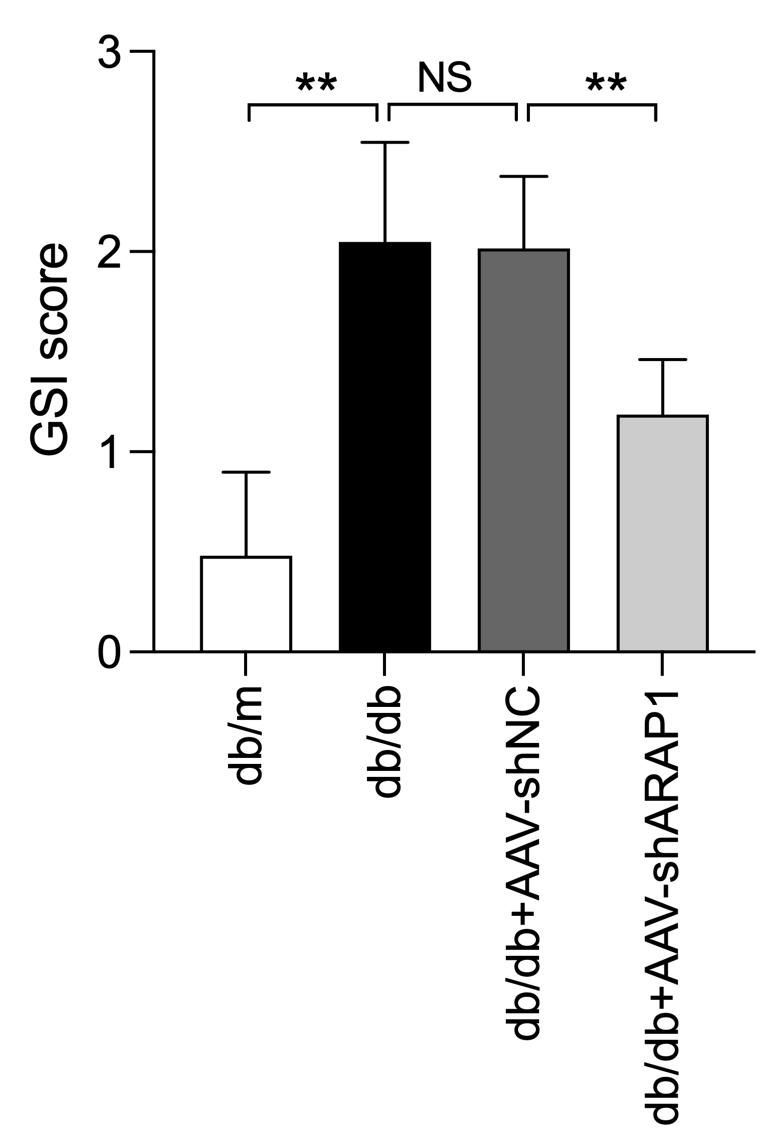


**Figure S5. Glomerulosclerosis index (GSI) score**

Glomerulosclerosis index (GSI) score of 30-50 glomeruli in diabetic (db/db) / normal

(db/m) mouse kidney tissue. Datas are presented as the mean±SD. **P*<0.05, ***P*<0.01, NS, not significant.


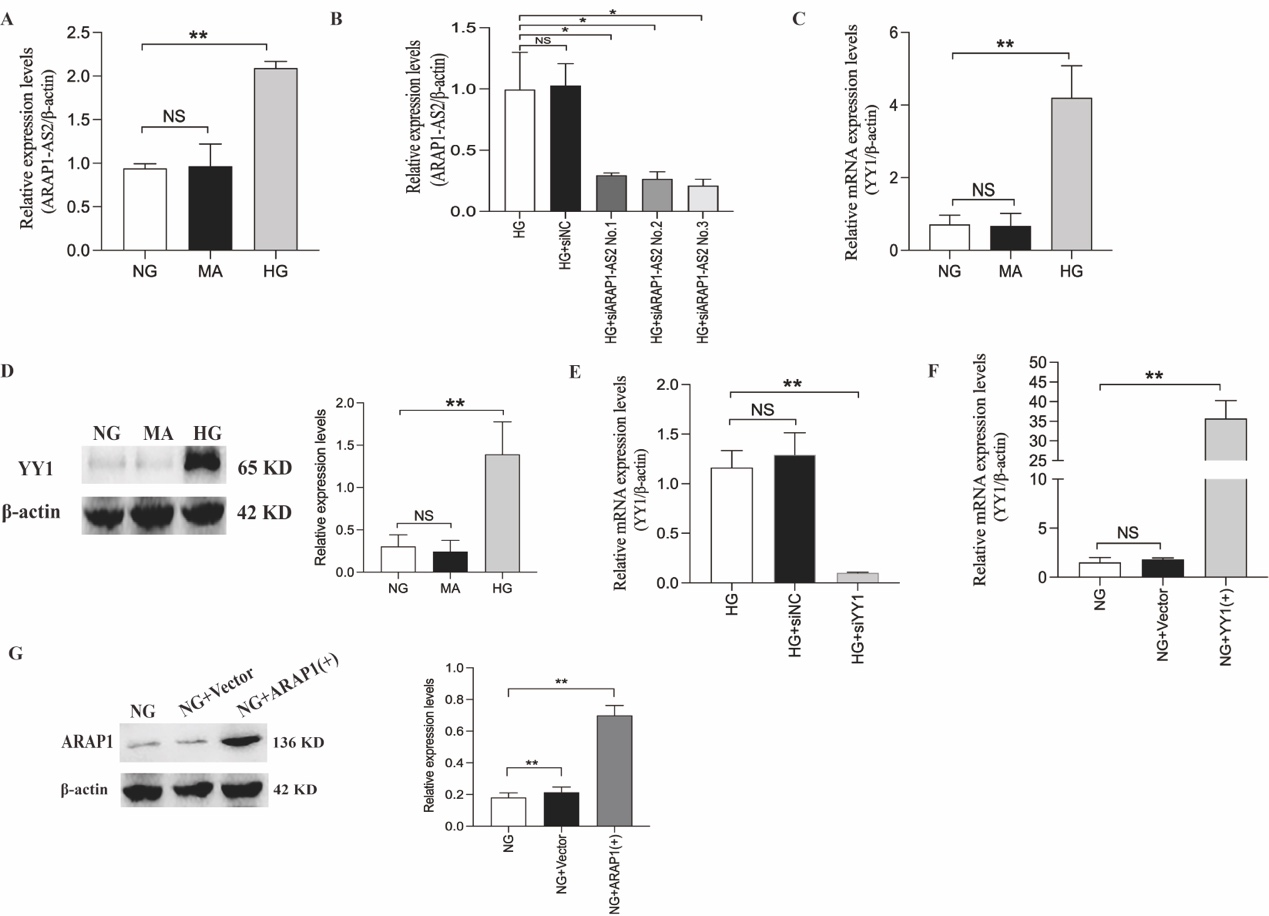


**Figure S6.** (A) qRT-PCR analysis of the expression of ARAP1-AS2 in HRMCs of the NG group and MA group and HG group. (B) Forty-eight hours after transfection of the ARAP1-AS2 siRNAs (50 nmol/L) in the HG group in 6-well plates, ARAP1-AS2 knockdown efficiencies were assessed by qRT-PCR and No.3 siRNA had the best knockdown effect. (C) The mRNA expression levels of YY1 in HRMCs of the NG group and MA group and HG group were detected by qRT-PCR. (D) The protein expression levels of YY1 in HRMCs of the NG group and MA group and HG group were detected by Western blot analysis. (E) Forty-eight hours after transfection of the YY1 siRNAs (50 nmol/L) in the HG group in 6-well plates, YY1 knockdown efficiencies were assessed by qRT-PCR. (F) Forty-eight hours after transfection of the YY1 overexpression plasmid (3000 ng) in the NG group in 6-well plates, YY1 overexpression efficiencies were assessed by qRT-PCR. (G) Forty-eight hours after transfection of the ARAP1 overexpression plasmid (3000 ng) in the NG group in 6-well plates, ARAP1 overexpression efficiencies were assessed by qRT-PCR. In all panels, the data are representative of three independent experiments. Datas are presented as the mean±SD. **P*<0.05, ***P*<0.01, NS, not significant.

**Supplementary materials and methods**

**1. Transfection and cell groups and naming**

All plasmids were transfected at an amount of 3000 ng, and all siRNAs were transfected with jetPRIME® at a 50 nmol/L final concentration following the manufacturer's protocol in the experiment. Cells in the HG group were transfected with human YY1 overexpression plasmid, human YY1 siRNA, human ARAP1-AS2 siRNA, human ARAP1 shRNA and mouse ARAP1 siRNA and named the HG+YY1(+), HG+siYY1, HG+siARAP1-AS2, HG+shARAP1 and HG+siARAP1 groups, respectively. Cells in the HG group transfected with the empty vector targeting human YY1, siRNANC targeting human YY1, human ARAP1-AS2 and mouse ARAP1, shNC targeting human ARAP1 were named the HG+Vector, HG+siNC and HG+shNC groups, respectively. Cells in the NG group were transfected with human YY1 overexpression plasmid, human YY1 siRNA, human ARAP1-AS2 siRNA, human ARAP1 overexpression plasmid and human ARAP1 shRNA and named the NG+YY1(+), NG+siYY1, NG+siARAP1-AS2, NG+ARAP1(+) and NG+shARAP1 groups, respectively. Cells in the NG group transfected with the empty vector targeting human YY1, siRNANC targeting human YY1, human ARAP1-AS2, shNC targeting human ARAP1 were named the NG+Vector, NG+siNC and NG+shNC groups, respectively.

**2. RNA pulldown sequencing (pulldown-seq)**

NEBNext Ultra II RNA Library Prep|NEB library construction kit was used to construct an RNA pull-down enrichment library for Illumina's high-throughput sequencing platform. HTSeq was used to calculate FPKM values, and we analyzed the differences between the input and the pull-down samples to identify lncRNA-bound mRNAs. Fold change value=Log_2_ ((FPKM in Positive+1)/(FPKM in Negative+1)).

**3. Animal groups and naming**

All mice were randomly assigned into four groups as follows: Group 1: diabetes group+ARAP1 shRNA AAV-2/9, to knockdown ARAP1 in kidney of diabetic db/db mice, we injected volume of 0.1 ml of AAV2/9 expressing ARAP1 shRNA (1×10^11^GC/animal) by tail vein at 13th week and named db/db+AAV-shARAP1 group (n=10). Group 2: diabetes group+empty viral vector, db/db mice received the same volume of 0.1 ml of AAV2/9-U6-NC (1×10^11^GC/animal) injection and named db/db+AAV-shNC group (n=10). Group 3: diabetes group, db/db mice injected with the equal volume of 0.1 ml of vehicle (PBS) and named db/db group (n=10). Group 4: control group, db/m mice were used as normal control and named db/m group (n=10). qRT-PCR and Western blot analysis was used to detect the transfection efficacy.

**4. GSI**

GSI was graded on a scale of 0 to 4 (0: normal; 1: involvement of <25% of the glomerulus, 2: involvement of 25–50% of the glomerulus; 3: involvement of 51–75% of the glomerulus and 4: involvement of >75% of the glomerulus).

**5. Isolation of mouse glomeruli**

Mice were anesthetized and perfused through the heart with 8×10^7^ Dynabeads M-450 (Thermo Scientific, Rockford, IL, USA) in PBS. Kidneys were minced into 1 mm^3^ and digested with collagenase A (1 mg/ml; Roche Diagnostics, Indianapolis, IN, USA) and deoxyribonuclease I (100 U/ml; Roche Diagnostics, Indianapolis, IN, USA) at 37 °C for 15 min, filtered with a 100um cell strainer, and the glomeruli containing Dynabeads were collected by a magnetic particle concentrator and washed three times with cold HBSS. The isolated glomeruli were lysed and followed subsequent analyses.

**6. IHC determination**

The deparaffinized and rehydrated sections (3 μm) were subjected to routine antigen retrieval, incubated with 3% H_2_O_2_ for 10 mins. After blocking with 5% BSA, these sections were incubated with the primary antibody overnight at 4°C and followed by incubation with the corresponding secondary antibody. The reaction was visualized with DAB solution. After nuclei were counterstained, the sections were sealed with neutral balsam and viewed under the light microscope (400×, Nikon, Japan).

**7. Immunofluorescence staining**

**7.1 For HRMC**

HRMC cells cultured with normal or high levels of glucose were fixed with 4% paraformaldehyde，blocked with goat serum and incubated with primary antibody overnight at 4°C. After washing with PBS, fluorescent-labeled secondary antibody was added. Nuclei were stained with DAPI. All images were observed with a fluorescence microscope and analysed with LAS AF Lite (Leica).

**7.2 For tissue sections**

The renal tissues sections were subjected to antigen retrieval, blocked with 3% hydrogen peroxide at room temperature, blocked with goat serum, and subsequently incubated with primary antibody at 4°C overnight. The sections were washed with PBS and incubated with corresponding fluorescent label-conjugated secondary antibody at room temperature. Nuclei were stained with DAPI and the sections were sealed by using the anti-fluorescence quencher. All images were observed with a fluorescence microscope and analysed with LAS AF Lite (Leica).

**8.qRT-PCR**

Total RNA was extracted from mouse glomeruli and cells using TRIzol reagent (Invitrogen, USA). The RNA was reverse transcribed to cDNA using Prime-Script^TM^ RT reagent Kit with gDNA Eraser (Takara, Japan). The mRNA expression levels were measured by TB Green^®^ Premix Ex Taq™ II (Takara, Japan) on a CFX96 PCR System (Bio-Rad, Hercules, CA). β-actin (also known as ACTB) expression was used as a control for normalization and relative quantitative data regarding mRNA levels were analysed by the 2^-ΔΔCt^ method.

**9.Western blot analysis**

Total protein from mouse glomeruli and cells were lysed in RIPA buffer. Equal amounts of protein were separated by SDS-PAGE and transferred to PVDF membranes (Millipore, USA). After being blocked in 5% BSA for 1.5 h, the membranes were incubated with primary antibody overnight at 4°C. The membranes were then incubated with HRP-conjugated secondary antibody at room temperature. Immunoreactive bands were visualized using ECL substrates (Millipore, USA). β-actin served as a loading control. ImageJ software was used to analyze the grey value of the protein bands.

**10. Coimmunoprecipitation (Co-IP)**

**For mouse glomeruli**

In brief, anti-ARAP1 (5ug, NB100-68223, Novus Biologicals) or anti-CIN85 (5ug, #12304, Cell Signaling Technology) antibody (Ab) or corresponding control rabbit IgG (5ug, B900610, Proteintech) was incubated with 50 μl of Dynabeads protein G for 2 h. Then we added Dynabeads-Ab complexes to equal amounts of mouse glomeruli protein lysates and incubated the mixtures overnight at 4°C with rotation. The Dynabeads–Ab–antigen complex was washed and subjected to Western blot analysis

**11. Ubiquitination assay**

**For mouse glomeruli**

The mouse glomeruli lysate was immunoprecipitated using Dynabeads protein A (Invitrogen, USA) using 10 μg rabbit anti-EGFR antibody (ab52894, Abcam) or rabbit IgG (10ug, B900610, Proteintech). Rabbit anti-EGFR (1:1000, ab52894, Abcam) and rabbit anti-ubiquitin (1:1000, #3933, Cell Signaling Technology) were used for Western blotting.
